# Supplementary material for: Orbitrap Collision Cross Section Measurements Enhance Isomer Annotations in Lipidomics
Source: bioRxiv. 2026 Jul 3:2026.07.03.735735. Preprint. [Version 1] doi: 10.64898/2026.07.03.735735 (PMC13345044; doi:10.64898/2026.07.03.735735)
Supplement: Supplement 1 [file media-1.zip › OrbiCCS_SI_070326.pdf]

Supplementary Information

Orbitrap Collision Cross Section Measurements Enhance Isomer Annotations in Lipidomics.

Ziqin (Grace) Ni[1], Konstantin Ayzikov[2], Alexander Makarov[2], Samuel Moore[3], David Gaul[1,3], Kyle Fort[2], Facundo M. Fernández[1,3*]

1. School of Chemistry and Biochemistry, Georgia Institute of Technology, Atlanta, Georgia 30332, United States
2. Thermo Fisher Scientific, Bremen, Germany
3. Petit Institute of Bioengineering and Bioscience, Georgia Institute of Technology, Atlanta, Georgia 30332, United States

[*]**Address correspondence to:** facundo.fernandez@chemistry.gatech.edu

Table of Contents

- Supplementary Table S1. Orbitrap resolution settings and corresponding transient durations.
- Supplementary Table S2. Summary of ^{Orbi}CCS values for 202 annotated lipid species in SRM1950 plasma.
- Supplementary Table S3. Summary of lipid features with matched ^{Orbi}CCS and ^{TIMS}CCS values from a single LC-Orbitrap experiment (pump speed 68%, resolution setting 240,000). The same dataset present in Fig. 3a-f.
- Supplementary Table S4. Summary of lipid features with matched ^{Orbi}CCS and ^{DT}CCS values from a single LC-Orbitrap experiment (pump speed 68%, resolution setting 240,000). The same dataset present in Fig. 3a-f.
- Supplementary Table S5. Isotopically labeled lipid standards.
- Supplementary Figure S6. Slope and intercept values used to calibrate ^{Orbi}CCS during LC-MS experiments.
- Supplementary Table S7. Summary of lipid features with matched ^{Orbi}CCS to reference ^{TIMS}CCS dataset for experiments conducted at a resolution of 240,000 and turbopump speed 68%.
- Supplementary Table S8. Summary of lipid features with matched ^{Orbi}CCS to reference ^{DT}CCS dataset for experiments conducted at a resolution of 240,000 and turbopump speed 68%.
- Supplementary Table S9. Summary of lipid features with matched ^{Orbi}CCS to reference ^{TIMS}CCS dataset for experiment conducted at resolution 180,000 and turbopump speed 68%.

- Supplementary Table S10. Summary of lipid features with matched ^{Orbi}CCS to reference ^{DT}CCS dataset for experiments conducted at a resolution of 180,000 and turbopump speed 68%.
- Supplementary Table S11. Summary of lipid features with matched ^{Orbi}CCS to reference ^{TIMS}CCS dataset for experiments conducted at a resolution of 120,000 and turbopump speed 68%.
- Supplementary Table S12. Summary of lipid features with matched ^{Orbi}CCS to reference ^{DT}CCS dataset for experiments conducted at a resolution of 120,000 and turbopump speed 68%.
- Supplementary Table S13. Summary of lipid features with matched ^{Orbi}CCS to reference ^{TIMS}CCS dataset for experiments conducted at a resolution of 90,000 and turbopump speed 68%.
- Supplementary Table S14. Summary of lipid features with matched ^{Orbi}CCS to reference ^{DT}CCS dataset for experiments conducted at a resolution of 90,000 and turbopump speed 68%.

Supplementary Table S1. Orbitrap resolution settings and corresponding transient durations. Summary of resolution settings used on the Thermo Fisher Orbitrap Exploris™ 240 mass spectrometer and the corresponding transient acquisition durations for a single scan.

**Resolution setting at *m/z* 200**	**Duration of transient signal (ms)**
60,000	128
90,000	192
120,000	256
180,000	384
240,000	512

Supplementary Table S2. **Summary of ^{Orbi}CCS of 202 annotated lipid species in IPA extract of SRM1950 plasma.** Table reports lipid class, proposed annotation, experimental monoisotopic m/z value, retention time, ^{Orbi}CCS values, and relevant statistics across four LC experiments conducted at 68% turbopump speed. Annotation was assigned based on the following criteria: 1) MS^1 and MS/MS spectra of the standard matched to the compound. 2) MS1 and MS/MS spectrum of the feature matched with library spectra.

#	Name	Formula	*m/z*	Type	RT1	RT2	RT3	RT4	CCS1	CCS2	CCS3	CCS4	meanCCS	stdCCS	RSD(%)
1	Car(5:0)	C12 H23 N O4	246.1699	Car	0.82	0.81	0.84	0.83	139.2	136.2	135.5	136.4	136.8	1.6	1.2
2	Car(10:0-OH)	C17 H33 N O5	314.2326	Car	0.92	0.92	0.93	0.94	163.0	159.7	161.2	160.9	161.2	1.3	0.8
3	Car(10:0)	C17 H33 N O4	316.2482	Car	0.98	0.98	1.00	1.01	163.0	159.8	161.0	160.8	161.2	1.3	0.8
4	Car(12:0)	C19 H37 N O4	344.2795	Car	1.21	1.21	1.22	1.23	172.5	171.6	171.8	172.1	172.0	0.4	0.2
5	Car(14:1)	C21 H39 N O4	370.2951	Car	1.37	1.36	1.38	1.39	182.2	181.2	182.3	180.1	181.4	1.1	0.6
6	Car(14:0)	C21 H41 N O4	372.3108	Car	1.60	1.59	1.63	1.65	183.6	182.8	183.1	180.9	182.6	1.2	0.6
7	Car(16:1)	C23 H43 N O4	398.3265	Car	1.72	1.71	1.76	1.78	192.8	193.0	191.8	190.3	192.0	1.3	0.7
8	Car(16:0)	C23 H45 N O4	400.3421	Car	2.01	2.01	2.04	2.05	190.7	188.6	189.7	187.2	189.1	1.5	0.8
9	Car(18:2)	C25 H45 N O4	424.3422	Car	1.82	1.82	1.87	1.88	201.7	199.4	200.1	199.4	200.2	1.1	0.5
10	Car(20:4)	C27 H45 N O4	448.3421	Car	1.79	1.79	1.84	1.86	211.0	211.3	210.8	210.3	210.8	0.4	0.2
11	CE(20:4)+NH3	C47 H79 N O2	690.6185	CE	8.91	8.91	8.91	8.92	281.8	280.7	283.8	285.7	283.0	2.2	0.8
12	CE(22:6)+NH3	C49 H79 N O2	714.6188	CE	8.70	8.70	8.70	8.70	286.8	287.3	291.4	291.1	289.2	2.4	0.8
13	LPC(14:0)>LPC(14:0/0:0) _and_LPC(0:0/14:0)	C22 H46 N O7 P	468.3085	LPC	1.62	1.62	1.66	1.67	215.1	214.2	213.9	212.4	213.9	1.1	0.5
14	LPC(14:0)>LPC(14:0/0:0) _and_LPC(0:0/14:0)	C22 H46 N O7 P	468.3085	LPC	1.52	1.52	1.55	1.57	215.4	214.5	214.0	212.5	214.1	1.2	0.6
15	LPC(15:0)>LPC(15:0/0:0) _and_LPC(0:0/15:0)	C23 H48 N O7 P	482.3242	LPC	1.76	1.76	1.81	1.82	220.9	220.7	223.3	220.4	221.3	1.3	0.6
16	LPC(16:1)>LPC(16:1/0:0) _and_LPC(0:0/16:1)	C24 H48 N O7 P	494.3242	LPC	1.64	1.64	1.68	1.69	223.3	222.1	223.3	222.1	222.7	0.7	0.3
17	LPC(16:0)>LPC(16:0/0:0) _and_LPC(0:0/16:0)	C24 H50 N O7 P	496.3396	LPC	2.08	2.07	2.10	2.11	221.7	220.5	221.3	219.9	220.8	0.8	0.4
18	LPC(16:0)>LPC(16:0/0:0) _and_LPC(0:0/16:0)	C24 H50 N O7 P	496.3398	LPC	2.76	2.76	2.77	2.77	221.2	220.7	219.3	218.2	219.8	1.4	0.6
19	LPC(16:0)>LPC(16:0/0:0) _and_LPC(0:0/16:0)	C24 H50 N O7 P	496.3399	LPC	1.99	1.99	2.02	2.03	221.7	220.4	221.8	220.5	221.1	0.8	0.3
20	LPC(17:0)>LPC(17:0/0:0) _and_LPC(0:0/17:0)	C25 H52 N O7 P	510.3555	LPC	2.25	2.25	2.26	2.22	226.4	225.4	225.9	223.1	225.2	1.5	0.6
21	LPC(18:3)>LPC(18:3/0:0) _and_LPC(0:0/18:3)	C26 H48 N O7 P	518.3242	LPC	1.64	1.64	1.68	1.65	230.4	230.0	231.6	230.8	230.7	0.7	0.3
22	LPC(20:5)>LPC(20:5/0:0) _and_LPC(0:0/20:5)	C28 H48 N O7 P	520.3397	LPC	1.86	1.86	1.82	1.84	229.3	229.2	231.2	230.1	230.0	0.9	0.4
23	LPC(18:1)>LPC(18:1/0:0) _and_LPC(0:0/18:1)	C26 H52 N O7 P	522.3552	LPC	2.07	2.07	2.09	2.11	229.2	229.2	230.7	229.7	229.7	0.7	0.3
24	LPC(18:1)>LPC(18:1/0:0) _and_LPC(0:0/18:1)	C26 H52 N O7 P	522.3554	LPC	2.14	2.14	2.16	2.17	229.2	229.4	230.3	229.3	229.5	0.5	0.2
25	LPC(18:0)>LPC(18:0/0:0) _and_LPC(0:0/18:0)	C26 H54 N O7 P	524.3710	LPC	2.39	2.40	2.40	2.40	229.2	229.8	229.8	228.9	229.4	0.5	0.2
26	LPC(18:0)>LPC(18:0/0:0) _and_LPC(0:0/18:0)	C26 H54 N O7 P	524.3713	LPC	2.76	2.73	2.77	2.77	229.3	229.9	229.2	228.4	229.2	0.6	0.3
27	LPC(19:1)>LPC(19:1/0:0) _and_LPC(0:0/19:1)	C27 H54 N O7 P	536.3712	LPC	2.29	2.29	2.30	2.31	238.2	237.6	239.2	239.0	238.5	0.8	0.3

#	Name	Formula	*m/z*	Type	RT1	RT2	RT3	RT4	CCS1	CCS2	CCS3	CCS4	meanCCS	stdCCS	RSD(%)
28	LPC(19:0)>LPC(19:0/0:0) _and_LPC(0:0/19:0)	C27 H56 N O7 P	538.3870	LPC	2.55	2.54	2.55	2.54	240.1	239.8	238.8	240.0	239.7	0.6	0.3
29	LPC(20:4)>LPC(20:4/0:0) _and_LPC(0:0/20:4)	C28 H50 N O7 P	544.3398	LPC	1.84	1.84	1.81	1.82	237.1	235.8	237.6	236.1	236.7	0.8	0.4
30	LPC(20:3)>LPC(20:3/0:0) _and_LPC(0:0/20:3)	C28 H52 N O7 P	546.3555	LPC	2.00	2.09	2.03	2.04	237.3	235.9	237.3	235.7	236.6	0.9	0.4
31	LPC(20:3)>LPC(20:3/0:0) _and_LPC(0:0/20:3)	C28 H52 N O7 P	546.3558	LPC	1.93	1.93	1.96	1.98	237.6	236.1	237.7	236.2	236.9	0.9	0.4
32	LPC(20:2)>LPC(20:2/0:0) _and_LPC(0:0/20:2)	C28 H54 N O7 P	548.3713	LPC	2.21	2.21	2.23	2.23	240.4	238.7	239.5	238.8	239.3	0.8	0.3
33	LPC(20:1)>LPC(20:1/0:0) _and_LPC(0:0/20:1)	C28 H56 N O7 P	550.3867	LPC	2.42	2.42	2.42	2.43	239.9	239.3	240.3	241.4	240.2	0.9	0.4
34	LPC(20:0)>LPC(20:0/0:0) _and_LPC(0:0/20:0)	C28 H58 N O7 P	552.4025	LPC	2.72	2.72	2.73	2.74	241.8	240.8	241.3	242.6	241.6	0.8	0.3
35	LPC(22:6)>LPC(22:6/0:0) _and_LPC(0:0/22:6)	C30 H50 N O7 P	568.3398	LPC	1.77	1.77	1.76	1.77	244.7	243.1	243.9	242.6	243.6	0.9	0.4
36	LPC(22:5)>LPC(22:5/0:0) _and_LPC(0:0/22:5)	C30 H52 N O7 P	570.3554	LPC	1.90	1.90	1.94	1.95	244.7	243.6	244.9	244.3	244.4	0.6	0.2
37	LPC(22:4)>LPC(22:4/0:0) _and_LPC(0:0/22:4)	C30 H54 N O7 P	572.3711	LPC	2.13	2.13	2.15	2.16	245.5	245.7	246.5	247.8	246.4	1.0	0.4
38	LPC(22:0)>LPC(22:0/0:0) _and_LPC(0:0/22:0)	C30 H62 N O7 P	580.4340	LPC	3.12	3.13	3.13	3.13	256.9	252.0	256.9	254.0	255.0	2.4	0.9
39	PC(28:0)	C36 H72 N O8 P	678.5070	PC	3.53	3.53	3.53	3.53	275.1	274.6	273.0	276.3	274.7	1.4	0.5
40	PC(O-30:0)	C38 H78 N O7 P	692.5586	PC	4.55	4.55	4.57	4.56	289.6	289.3	282.9	282.3	286.0	3.9	1.4
41	PC(30:1)	C38 H74 N O8 P	704.5226	PC	3.58	3.58	3.59	3.59	281.2	283.1	281.9	282.0	282.1	0.8	0.3
42	PC(30:0)	C38 H76 N O8 P	706.5380	PC	4.10	4.10	4.10	4.09	279.1	280.0	278.4	278.1	278.9	0.8	0.3
43	PC(O-32:2)	C40 H78 N O7 P	716.5583	PC	4.54	4.53	4.54	4.53	284.6	284.5	282.7	282.3	283.5	1.2	0.4
44	PC(31:1)	C39 H76 N O8 P	718.5383	PC	3.86	3.86	3.87	3.88	292.1	291.8	286.4	285.3	288.9	3.5	1.2
45	PC(O-32:1)	C40 H80 N O7 P	718.5748	PC	5.26	5.25	5.27	5.27	283.4	283.5	281.4	281.8	282.5	1.1	0.4
46	PC(O-32:1)	C40 H80 N O7 P	718.5751	PC	4.66	4.66	4.68	4.66	285.8	285.1	280.7	282.3	283.5	2.4	0.8
47	PC(O-32:0)	C40 H82 N O7 P	720.5905	PC	5.38	5.37	5.40	5.38	283.8	284.3	281.6	281.7	282.9	1.4	0.5
48	PC(32:2)	C40 H76 N O8 P	730.5384	PC	3.70	3.69	3.70	3.70	284.0	285.4	283.9	283.4	284.2	0.9	0.3
49	PC(32:1)	C40 H78 N O8 P	732.5540	PC	4.18	4.18	4.18	4.17	284.4	285.3	283.7	283.4	284.2	0.9	0.3
50	PC(32:0)	C40 H80 N O8 P	734.5698	PC	4.83	4.82	4.82	4.82	283.4	283.8	282.9	283.0	283.3	0.4	0.1
51	PC(33:2)	C41 H78 N O8 P	744.5543	PC	4.00	4.00	4.01	4.01	288.8	290.0	288.4	288.1	288.8	0.8	0.3
52	PC(O-34:2)	C42 H82 N O7 P	744.5898	PC	5.32	5.32	5.33	5.32	289.0	289.7	288.1	288.3	288.8	0.7	0.3
53	PC(O-34:2)	C42 H82 N O7 P	744.5905	PC	4.83	4.82	4.82	4.82	292.2	291.4	285.4	285.0	288.5	3.8	1.3
54	PC(33:1)	C41 H80 N O8 P	746.5697	PC	4.52	4.52	4.53	4.53	285.9	287.6	286.8	287.2	286.9	0.7	0.3
55	PC(O-34:1)	C42 H84 N O7 P	746.6063	PC	5.44	5.43	5.45	5.46	289.9	290.8	288.2	287.9	289.2	1.4	0.5
56	PC(O-34:0)	C42 H86 N O7 P	748.6221	PC	6.31	6.31	6.31	6.30	291.5	293.0	289.4	288.7	290.6	1.9	0.7
57	PC(34:4)	C42 H76 N O8 P	754.5384	PC	3.62	3.61	3.62	3.62	292.8	292.5	290.7	290.0	291.5	1.4	0.5
58	PC(34:3)	C42 H78 N O8 P	756.5539	PC	3.77	3.76	3.77	3.78	292.6	292.5	291.0	290.2	291.6	1.2	0.4
59	PC(34:3)	C42 H78 N O8 P	756.5540	PC	3.89	3.89	3.90	3.90	292.6	292.4	291.2	290.2	291.6	1.1	0.4
60	PC(34:2)	C42 H80 N O8 P	758.5693	PC	4.32	4.32	4.32	4.33	290.2	290.0	290.4	290.7	290.3	0.3	0.1
61	PC(34:1)	C42 H82 N O8 P	760.5850	PC	4.89	4.89	4.88	4.88	293.1	293.1	293.4	292.9	293.1	0.2	0.1
62	PC(34:0)	C42 H84 N O8 P	762.6010	PC	5.70	5.70	5.69	5.69	297.3	297.8	293.5	290.6	294.8	3.4	1.2
63	PC(O-36:5)	C44 H80 N O7 P	766.5748	PC	4.23	4.23	4.23	4.21	320.3	300.3	307.2	307.0	308.7	8.4	2.7
64	PC(35:4)	C43 H78 N O8 P	768.5538	PC	5.19	5.19	5.20	5.20	296.5	297.6	294.4	293.4	295.5	1.9	0.7
65	PC(35:4)	C43 H78 N O8 P	768.5540	PC	3.89	3.89	3.90	3.90	296.7	296.9	294.1	293.0	295.2	1.9	0.6

#	Name	Formula	*m/z*	Type	RT1	RT2	RT3	RT4	CCS1	CCS2	CCS3	CCS4	meanCCS	stdCCS	RSD(%)
66	PC(O-36:4)	C44 H82 N O7 P	768.5903	PC	4.69	4.69	4.70	4.70	294.1	294.8	292.6	290.7	293.0	1.8	0.6
67	PC(35:3)	C43 H80 N O8 P	770.5691	PC	4.05	4.05	4.05	4.04	298.9	300.5	295.3	294.7	297.3	2.8	0.9
68	PC(35:3)	C43 H80 N O8 P	770.5695	PC	4.17	4.16	4.16	4.15	299.0	300.5	294.7	294.7	297.2	3.0	1.0
69	PC(O-36:3)	C44 H84 N O7 P	770.6058	PC	5.03	5.02	5.03	5.03	296.6	296.5	294.2	292.4	294.9	2.0	0.7
70	PC(35:2)	C43 H82 N O8 P	772.5850	PC	4.57	4.57	4.58	4.58	293.2	293.2	292.5	292.5	292.9	0.4	0.1
71	PC(O-36:2)	C44 H86 N O7 P	772.6216	PC	5.72	5.71	5.71	5.70	299.5	300.0	296.3	291.4	296.8	3.9	1.3
72	PC(O-36:2)	C44 H86 N O7 P	772.6217	PC	6.22	6.22	6.22	6.22	300.0	299.8	295.5	290.9	296.6	4.3	1.4
73	PC(35:1)	C43 H84 N O8 P	774.6007	PC	5.19	5.19	5.18	5.19	303.7	305.9	293.1	293.0	298.9	6.8	2.3
74	PC(35:1)	C43 H84 N O8 P	774.6008	PC	5.32	5.32	5.33	5.32	304.4	306.3	293.4	292.8	299.3	7.1	2.4
75	PC(O-36:1)	C44 H88 N O7 P	774.6370	PC	6.37	6.37	6.36	6.36	300.9	301.8	293.3	283.0	294.8	8.7	3.0
76	PC(36:6)	C44 H76 N O8 P	778.5382	PC	3.53	3.52	3.53	3.50	297.2	297.6	295.0	293.9	295.9	1.8	0.6
77	PC(36:6)	C44 H76 N O8 P	778.5384	PC	3.36	3.36	3.36	3.36	297.3	297.5	294.6	293.7	295.8	1.9	0.6
78	PC(36:5)	C44 H78 N O8 P	780.5538	PC	3.68	3.68	3.69	3.68	297.1	298.1	295.5	294.3	296.2	1.7	0.6
79	PC(36:5)	C44 H78 N O8 P	780.5539	PC	3.82	3.82	3.83	3.83	297.0	297.9	295.7	294.4	296.2	1.5	0.5
80	PC(O-37:5)	C45 H82 N O7 P	780.5900	PC	6.48	6.47	6.47	6.47	298.2	299.3	301.6	298.6	299.4	1.5	0.5
81	PC(O-37:5)	C45 H82 N O7 P	780.5903	PC	6.21	6.21	6.22	6.22	298.5	299.4	301.8	299.0	299.7	1.4	0.5
82	PC(36:4)	C44 H80 N O8 P	782.5693	PC	3.88	3.88	3.89	3.88	297.1	298.1	295.8	294.5	296.4	1.6	0.5
83	PC(36:4)	C44 H80 N O8 P	782.5694	PC	4.21	4.22	4.22	4.21	296.8	297.3	295.1	294.4	295.9	1.4	0.5
84	PC(36:3)	C44 H82 N O8 P	784.5848	PC	4.38	4.39	4.39	4.38	293.1	294.3	294.8	294.4	294.2	0.7	0.2
85	PC(36:3)	C44 H82 N O8 P	784.5851	PC	4.51	4.51	4.52	4.54	294.6	295.8	294.4	293.6	294.6	0.9	0.3
86	PC(36:2)	C44 H84 N O8 P	786.6007	PC	5.12	5.11	5.12	5.11	295.8	297.1	295.5	294.2	295.6	1.2	0.4
87	PC(36:2)	C44 H84 N O8 P	786.6007	PC	4.96	4.96	4.95	4.96	295.0	296.0	295.2	294.2	295.1	0.7	0.2
88	PC(36:1)	C44 H86 N O8 P	788.6165	PC	5.77	5.77	5.77	5.77	298.6	300.1	297.1	294.3	297.5	2.5	0.8
89	PC(37:6)	C45 H78 N O8 P	792.5536	PC	3.76	3.76	3.76	3.75	300.9	301.9	302.0	298.3	300.8	1.7	0.6
90	PC(O-38:5)	C46 H84 N O7 P	794.6058	PC	4.72	4.73	4.73	4.73	300.5	301.8	297.6	296.6	299.1	2.4	0.8
91	PC(O-38:5)	C46 H84 N O7 P	794.6059	PC	5.39	5.40	5.40	5.41	301.6	303.2	299.2	297.7	300.4	2.5	0.8
92	PC(O-38:4)	C46 H86 N O7 P	796.6217	PC	5.57	5.57	5.57	5.57	304.1	306.7	299.4	297.8	302.0	4.1	1.4
93	PC(37:3)	C45 H84 N O8 P	798.6002	PC	4.90	4.90	4.89	4.91	303.8	303.5	300.8	297.0	301.3	3.1	1.0
94	PC(O-38:3)	C46 H88 N O7 P	798.6372	PC	5.92	5.92	5.94	5.93	319.1	319.9	299.8	297.0	308.9	12.3	4.0
95	PC(37:1)	C45 H88 N O8 P	802.6322	PC	6.20	6.21	6.22	6.22	307.1	306.4	305.3	303.1	305.5	1.8	0.6
96	PC(38:7)	C46 H78 N O8 P	804.5538	PC	3.55	3.55	3.56	3.56	304.8	305.6	301.6	300.6	303.1	2.4	0.8
97	PC(38:7)	C46 H78 N O8 P	804.5539	PC	3.45	3.45	3.46	3.46	304.9	305.5	301.3	300.5	303.1	2.5	0.8
98	PC(38:6)	C46 H80 N O8 P	806.5694	PC	4.06	4.06	4.06	4.05	304.4	305.7	302.1	301.0	303.3	2.1	0.7
99	PC(38:6)	C46 H80 N O8 P	806.5695	PC	3.79	3.78	3.80	3.79	304.3	305.6	302.0	301.0	303.2	2.1	0.7
100	PC(38:4)	C46 H84 N O8 P	810.6006	PC	4.56	4.57	4.57	4.58	302.5	304.1	300.6	300.4	301.9	1.7	0.6
101	PC(38:4)	C46 H84 N O8 P	810.6008	PC	5.00	5.00	4.99	4.98	302.4	303.7	301.3	300.6	302.0	1.4	0.5
102	PC(38:4)	C46 H84 N O8 P	810.6008	PC	4.72	4.73	4.73	4.73	303.0	304.7	300.6	300.0	302.1	2.2	0.7
103	PC(38:3)	C46 H86 N O8 P	812.6165	PC	5.33	5.33	5.34	5.35	304.8	307.1	303.3	301.6	304.2	2.3	0.8
104	PC(38:2)	C46 H88 N O8 P	814.6321	PC	5.89	5.89	5.90	5.89	307.2	307.9	304.9	302.8	305.7	2.3	0.8
105	PC(38:1)	C46 H90 N O8 P	816.6475	PC	6.51	6.51	6.52	6.51	307.2	307.7	306.8	306.9	307.2	0.4	0.1
106	PC(O-40:7)	C48 H84 N O7 P	818.6052	PC	4.52	4.52	4.54	4.53	311.3	312.0	307.3	304.7	308.8	3.4	1.1
107	PC(O-40:7)	C48 H84 N O7 P	818.6057	PC	5.17	5.18	5.18	5.19	312.1	313.2	308.5	304.9	309.7	3.8	1.2
108	PC(39:6)	C47 H82 N O8 P	820.5850	PC	4.41	4.41	4.42	4.42	307.5	308.1	309.5	307.7	308.2	0.9	0.3
109	PC(O-40:6)	C48 H86 N O7 P	820.6210	PC	5.32	5.33	5.34	5.35	314.2	315.0	309.2	305.1	310.9	4.6	1.5
110	PC(39:4)	C47 H86 N O8 P	824.6166	PC	5.43	5.44	5.45	5.44	310.3	310.1	310.2	307.4	309.5	1.4	0.5
111	PC(O-40:4)	C48 H90 N O7 P	824.6524	PC	6.45	6.45	6.45	6.44	321.0	321.5	311.4	305.3	314.8	7.8	2.5
112	PC(40:8)	C48 H80 N O8 P	830.5695	PC	3.67	3.67	3.68	3.67	312.2	312.5	309.1	307.1	310.2	2.6	0.8
113	PC(40:7)	C48 H82 N O8 P	832.5845	PC	4.19	4.19	4.19	4.20	312.5	312.6	308.9	307.5	310.3	2.6	0.8

#	Name	Formula	*m/z*	Type	RT1	RT2	RT3	RT4	CCS1	CCS2	CCS3	CCS4	meanCCS	stdCCS	RSD(%)
114	PC(40:7)	C48 H82 N O8 P	832.5849	PC	4.12	4.11	4.11	4.10	312.5	312.8	309.5	307.3	310.5	2.6	0.9
115	PC(40:7)	C48 H82 N O8 P	832.5849	PC	3.93	3.93	3.92	3.94	312.5	313.0	309.4	307.4	310.6	2.6	0.8
116	PC(40:6)	C48 H84 N O8 P	834.6007	PC	4.80	4.79	4.80	4.79	311.6	312.0	307.4	306.4	309.3	2.8	0.9
117	PC(42:7)	C50 H86 N O8 P	838.6320	PC	5.60	5.59	5.59	5.58	313.9	314.8	309.9	308.3	311.7	3.1	1.0
118	PC(42:4)	C50 H92 N O8 P	848.6524	PC	6.24	6.24	6.25	6.24	319.3	319.4	316.6	313.1	317.1	2.9	0.9
119	PC(O-42:6)	C50 H90 N O7 P	848.6525	PC	5.67	5.66	5.66	5.65	318.7	319.9	317.5	313.8	317.5	2.6	0.8
120	PC(42:8)	C50 H84 N O8 P	858.6008	PC	4.12	4.12	4.11	4.10	319.7	322.0	319.8	320.2	320.4	1.1	0.3
121	PC(42:5)	C50 H90 N O8 P	864.6475	PC	5.83	5.83	5.83	5.83	323.7	325.6	322.8	318.3	322.6	3.1	1.0
122	PC(42:4)	C50 H92 N O8 P	866.6639	PC	6.63	6.63	6.63	6.63	323.5	325.0	323.1	320.3	323.0	2.0	0.6
123	PE(O-36:3)>PE(O-18:1/18:2)	C41 H78 N O7 P	728.5593	PE	5.60	5.60	5.60	5.59	288.4	288.0	288.0	286.5	287.7	0.8	0.3
124	PE(O-38:6)>PE(O-18:2/20:4)_and_PE(O-16:1/22:5)	C43 H76 N O7 P	750.5431	PE	4.68	4.68	4.69	4.68	293.6	294.9	293.7	290.2	293.1	2.0	0.7
125	PE(O-40:4)	C45 H84 N O7 P	782.6045	PE	5.11	5.09	5.11	5.11	295.7	295.4	308.9	299.9	300.0	6.3	2.1
126	SM(d32:2)	C37 H73 N2 O6 P	673.5281	SM	3.09	3.08	3.10	3.10	272.0	271.6	270.9	271.8	271.6	0.5	0.2
127	SM(d32:1)	C37 H75 N2 O6 P	675.5438	SM	3.49	3.48	3.49	3.50	272.1	271.5	271.0	272.3	271.7	0.6	0.2
128	SM(d32:0)	C37 H77 N2 O6 P	677.5598	SM	3.69	3.69	3.69	3.70	279.4	278.2	273.4	274.2	276.3	2.9	1.1
129	SM(d33:1)	C38 H77 N2 O6 P	689.5595	SM	3.76	3.76	3.76	3.76	275.5	277.3	275.2	276.0	276.0	0.9	0.3
130	SM(d34:2)	C39 H77 N2 O6 P	701.5595	SM	3.57	3.57	3.58	3.58	277.2	278.1	276.8	276.9	277.2	0.6	0.2
131	SM(d34:1)	C39 H79 N2 O6 P	703.5749	SM	4.06	4.07	4.06	4.06	276.8	277.6	276.7	276.6	276.9	0.4	0.2
132	SM(d34:0)	C39 H81 N2 O6 P	705.5905	SM	4.31	4.32	4.32	4.33	281.2	281.2	279.7	279.5	280.4	1.0	0.3
133	SM(d35:2)	C40 H79 N2 O6 P	715.5749	SM	3.86	3.86	3.84	3.83	288.6	286.7	285.3	283.5	286.0	2.1	0.8
134	SM(d35:1)	C40 H81 N2 O6 P	717.5906	SM	4.43	4.43	4.44	4.43	282.5	283.3	282.2	283.5	282.9	0.6	0.2
135	SM(d35:1)	C40 H81 N2 O6 P	717.5908	SM	4.26	4.27	4.27	4.28	297.4	298.6	287.4	291.4	293.7	5.3	1.8
136	SM(d34:0-OH)	C39 H81 N2 O7 P	721.5858	SM	3.74	3.74	3.76	3.75	285.9	287.1	286.3	286.3	286.4	0.5	0.2
137	SM(d36:3)	C41 H79 N2 O6 P	727.5753	SM	3.72	3.72	3.72	3.72	284.3	285.3	286.0	285.7	285.3	0.7	0.3
138	SM(d36:2)	C41 H81 N2 O6 P	729.5906	SM	4.19	4.19	4.19	4.19	285.1	285.7	285.3	285.4	285.4	0.2	0.1
139	SM(d36:1)	C41 H83 N2 O6 P	731.6065	SM	4.81	4.81	4.81	4.81	284.0	284.2	284.3	284.1	284.1	0.1	0.0
140	SM(d36:0)	C41 H85 N2 O6 P	733.6220	SM	5.12	5.11	5.12	5.11	294.4	293.3	287.8	289.9	291.4	3.0	1.0
141	SM(d37:1)	C42 H85 N2 O6 P	745.6220	SM	5.27	5.28	5.30	5.29	291.7	293.9	286.7	290.8	290.8	3.0	1.0
142	SM(d38:1)	C43 H87 N2 O6 P	759.6376	SM	5.77	5.77	5.77	5.77	291.9	292.9	293.9	292.3	292.7	0.9	0.3
143	SM(d39:1)	C44 H89 N2 O6 P	773.6532	SM	6.22	6.21	6.23	6.23	294.9	296.5	295.7	294.7	295.4	0.8	0.3
144	SM(d40:2)	C45 H89 N2 O6 P	785.6533	SM	5.69	5.69	5.68	5.68	300.8	301.7	300.3	298.8	300.4	1.2	0.4
145	SM(d40:2)	C45 H89 N2 O6 P	785.6534	SM	5.90	5.90	5.91	5.91	301.0	301.6	299.9	298.5	300.3	1.4	0.5
146	SM(d40:1)	C45 H91 N2 O6 P	787.6688	SM	6.53	6.53	6.53	6.54	302.7	302.4	299.5	298.5	300.8	2.1	0.7
147	SM(d41:2)	C46 H91 N2 O6 P	799.6690	SM	6.36	6.35	6.35	6.34	303.9	303.6	302.0	301.1	302.6	1.3	0.4
148	SM(d41:2)	C46 H91 N2 O6 P	799.6690	SM	6.09	6.10	6.11	6.10	303.9	303.7	302.2	301.4	302.8	1.2	0.4
149	SM(d41:1)	C46 H93 N2 O6 P	801.6847	SM	6.75	6.75	6.75	6.75	303.9	304.1	301.5	300.4	302.5	1.8	0.6
150	SM(d42:3)	C47 H91 N2 O6 P	811.6686	SM	5.79	5.80	5.80	5.80	307.4	308.7	306.9	304.6	306.9	1.7	0.6
151	SM(d42:2)	C47 H93 N2 O6 P	813.6845	SM	6.47	6.47	6.46	6.46	308.6	309.2	306.8	304.5	307.3	2.1	0.7
152	SM(d43:3)	C48 H93 N2 O6 P	825.6845	SM	6.23	6.24	6.24	6.24	310.7	311.9	309.7	308.3	310.2	1.5	0.5
153	SM(d43:2)	C48 H95 N2 O6 P	827.7000	SM	6.69	6.70	6.70	6.70	311.2	313.8	310.2	308.1	310.8	2.4	0.8
154	SM(d43:2)	C48 H95 N2 O6 P	827.7001	SM	6.82	6.81	6.82	6.81	311.3	313.7	309.9	307.8	310.7	2.5	0.8
155	SM(t42:1)_or_SM(d42:1-OH)	C47 H95 N2 O7 P	831.6951	SM	6.07	6.07	6.08	6.08	314.2	315.0	311.6	310.8	312.9	2.0	0.6
156	SM(d44:3)	C49 H95 N2 O6 P	839.6997	SM	6.56	6.56	6.57	6.56	314.7	315.6	314.0	312.4	314.2	1.3	0.4

#	Name	Formula	*m/z*	Type	RT1	RT2	RT3	RT4	CCS1	CCS2	CCS3	CCS4	meanCCS	stdCCS	RSD(%)
157	TG(44:1)>TG(10:0_16:0_18:1) + NH3	C47 H91 N O6	766.6921	TG	7.76	7.75	7.76	7.76	295.2	296.3	298.1	296.8	296.6	1.2	0.4
158	TG(46:2) + NH3	C49 H93 N O6	792.7076	TG	7.79	7.79	7.79	7.79	301.6	302.3	304.2	302.8	302.7	1.1	0.4
159	TG(46:1)>TG(12:0_16:0_18:1)_and_TG(10:0_18:0_18:1)_and_TG(14:0_16:0_16:1) + NH3	C49 H95 N O6	794.7235	TG	8.07	8.07	8.07	8.07	300.7	302.1	304.4	305.2	303.1	2.1	0.7
160	TG(46:0)>TG(14:0_16:0_16:0)_and_TG(12:0_16:0_18:0) + NH3	C49 H97 N O6	796.7390	TG	8.44	8.43	8.44	8.44	309.3	309.5	305.9	307.9	308.1	1.7	0.5
161	TG(48:3) + NH3	C51 H95 N O6	818.7235	TG	7.80	7.80	7.80	7.81	308.3	309.7	308.6	307.4	308.5	0.9	0.3
162	TG(48:2)>TG(14:0_16:0_18:2)_and_TG(14:0_16:1_18:1) + NH3	C51 H97 N O6	820.7390	TG	8.11	8.11	8.11	8.10	308.6	309.3	309.0	309.6	309.1	0.4	0.1
163	TG(48:1)>TG(16:0_16:0_16:1)_and_TG(14:0_16:0_18:1) + NH3	C51 H99 N O6	822.7546	TG	8.42	8.42	8.43	8.42	308.9	309.4	308.8	309.9	309.2	0.5	0.2
164	TG(49:3) + NH3	C52 H97 N O6	832.7390	TG	7.95	7.95	7.96	7.96	312.3	315.1	311.5	308.1	311.8	2.9	0.9
165	TG(49:1)>TG(15:0_16:0_18:0)_and_TG(16:0_16:0_17:1)_and_TG(16:0_16:1_17:0) + NH3	C52 H101 N O6	836.7702	TG	8.61	8.61	8.62	8.61	316.2	319.5	316.1	311.3	315.8	3.4	1.1
166	TG(50:4)>TG(16:1_16:1_18:2)_and_TG(16:1_16:1_18:2) + NH3	C53 H97 N O6	844.7388	TG	7.86	7.84	7.85	7.86	314.2	315.4	315.0	312.4	314.2	1.3	0.4
167	TG(50:3) + NH3	C53 H99 N O6	846.7545	TG	8.13	8.13	8.12	8.12	314.2	316.2	314.8	313.4	314.7	1.2	0.4
168	TG(50:2)>TG(16:0_16:1_18:1)_and_TG(14:0_18:1_18:1) + NH3	C53 H101 N O6	848.7701	TG	8.42	8.42	8.42	8.42	314.2	317.4	315.3	314.7	315.4	1.4	0.4
169	TG(50:1)>TG(16:0_16:0_18:1) + NH3	C53 H103 N O6	850.7858	TG	8.81	8.81	8.81	8.81	315.2	316.8	316.8	316.8	316.4	0.8	0.2
170	TG(51:4)>TG(16:1_17:1_18:2)_and_TG(15:0_18:2_18:2)_and_TG(15:1_18:1_18:2) + NH3	C54 H99 N O6	858.7545	TG	8.00	8.01	8.01	8.01	317.7	319.8	320.0	317.7	318.8	1.3	0.4
171	TG(51:2) M + NH3	C54 H103 N O6	862.7854	TG	7.79	7.79	7.79	7.79	318.9	320.0	320.9	319.6	319.9	0.8	0.3
172	TG(51:2)>TG(15:0_18:1_18:1)_and_TG(16:0_17:1_18:1)_and_TG(16:1_17:0_18:1) + NH3	C54 H103 N O6	862.7858	TG	8.60	8.60	8.60	8.60	318.8	320.2	320.2	320.2	319.8	0.7	0.2
173	TG(52:6) M + NH3	C55 H97 N O6	868.7390	TG	7.65	7.65	7.74	7.74	319.6	319.4	320.2	316.7	319.0	1.6	0.5
174	TG(52:5)>TG(16:0_18:2_18:3) + NH3	C55 H99 N O6	870.7545	TG	7.93	7.94	7.94	7.94	319.6	320.4	320.2	317.4	319.4	1.4	0.4
175	TG(52:4)>TG(16:0_16:0_20:4)_and_TG(16:1_18:1_18:2)_and_TG(16:0_18:1_18:2) + NH3	C55 H101 N O6	872.7702	TG	8.18	8.18	8.17	8.18	319.8	320.8	320.1	317.9	319.7	1.2	0.4
176	TG(52:3)>TG(16:0_18:1_18:2)_and_TG(16:1_18:1_18:1) + NH3	C55 H103 N O6	874.7858	TG	8.48	8.47	8.48	8.48	320.6	321.5	320.5	318.3	320.2	1.3	0.4

#	Name	Formula	*m/z*	Type	RT1	RT2	RT3	RT4	CCS1	CCS2	CCS3	CCS4	meanCCS	stdCCS	RSD(%)
177	TG(52:2)>TG(16:0_18:1_18:1) + NH3	C55 H105 N O6	876.8014	TG	7.79	7.80	7.80	7.80	337.4	337.1	336.8	320.4	333.0	8.3	2.5
178	TG(52:2)>TG(16:0_18:1_18:1) + NH3	C55 H105 N O6	876.8015	TG	8.80	8.79	8.80	8.79	320.5	322.4	321.0	319.1	320.8	1.4	0.4
179	TG(53:4)>TG(17:0_18:2_18:2)_and_TG(17:1_18:1_18:2) + NH3	C56 H103 N O6	886.7856	TG	8.30	8.30	8.30	8.30	327.2	329.7	328.8	324.6	327.6	2.2	0.7
180	TG(53:3)>TG(17:0_18:1_18:2)_and_TG(17:1_18:1_18:1)_and_TG(16:1_18:1_19:1)_and_TG(16:0_18:1_19:2) + NH3	C56 H105 N O6	888.8013	TG	7.80	7.80	7.81	7.81	328.0	330.1	329.6	324.3	328.0	2.7	0.8
181	TG(53:3)>TG(17:0_18:1_18:2)_and_TG(17:1_18:1_18:1)_and_TG(16:1_18:1_19:1)_and_TG(16:0_18:1_19:2) + NH3	C56 H105 N O6	888.8014	TG	8.66	8.66	8.67	8.66	327.9	330.2	328.8	324.8	327.9	2.3	0.7
182	TG(53:2) + NH3	C56 H107 N O6	890.8169	TG	8.82	8.81	8.81	8.80	331.6	334.1	331.6	332.4	332.4	1.2	0.3
183	TG(53:2) + NH3	C56 H107 N O6	890.8170	TG	8.99	8.99	9.00	9.00	331.6	333.9	332.1	331.9	332.4	1.0	0.3
184	TG(54:7)>TG(16:0_16:1_22:6)_and_TG(16:0_18:2_20:5) + NH3	C57 H99 N O6	894.7543	TG	7.84	7.84	7.85	7.84	326.2	328.1	328.5	322.9	326.4	2.6	0.8
185	TG(54:6)>TG(16:0_18:2_20:4) + NH3	C57 H101 N O6	896.7701	TG	8.07	8.06	8.06	8.07	326.8	329.5	329.5	323.6	327.4	2.8	0.9
186	TG(54:5)>TG(18:1_18:2_18:2)_and_TG(16:0_16:0_22:5)_and_TG(16:0_18:1_20:4) + NH3	C57 H103 N O6	898.7857	TG	8.36	8.35	8.36	8.36	327.4	329.9	329.5	324.0	327.7	2.7	0.8
187	TG(54:4)>TG(18:1_18:1_18:2) + NH3	C57 H105 N O6	900.8012	TG	8.47	8.46	8.47	8.47	329.0	331.5	329.9	324.4	328.7	3.0	0.9
188	TG(54:3)>TG(18:0_18:1_18:2)_and_TG(18:1/18:1/18:1)_and_TG(16:0_18:2_20:1) + NH3	C57 H107 N O6	902.8169	TG	8.87	8.87	8.87	8.87	330.3	331.8	329.7	324.7	329.1	3.1	0.9
189	TG(54:2)>TG(16:0_18:1_20:1)_and_TG(18:0_18:1_18:1) + NH3	C57 H109 N O6	904.8326	TG	9.20	9.20	9.20	9.20	334.5	335.1	334.8	331.2	333.9	1.8	0.5
190	TG(55:4)>TG(18:1_18:2_19:1)_and_TG(18:2_18:2_19:0)_and_TG(18:1_18:1_19:2) + NH3	C58 H107 N O6	914.8167	TG	7.85	7.83	7.85	7.86	342.9	344.0	342.1	335.8	341.2	3.7	1.1
191	TG(55:4)>TG(18:1_18:2_19:1)_and_TG(18:2_18:2_19:0)_and_TG(18:1_18:1_19:2) + NH3	C58 H107 N O6	914.8171	TG	8.65	8.65	8.65	8.65	343.0	344.1	341.5	336.4	341.2	3.4	1.0
192	TG(55:3)>TG(18:1_18:1_19:1)_and_TG(18:1_18:2_19:0) + NH3	C58 H109 N O6	916.8328	TG	8.97	8.97	8.98	8.98	343.9	344.6	342.0	336.3	341.7	3.7	1.1
193	TG(55:2)>TG(18:1_18:1_19:0) + NH3	C58 H111 N O6	918.8513	TG	8.81	8.82	8.81	8.81	344.0	344.9	343.0	337.3	342.3	3.4	1.0

#	Name	Formula	*m/z*	Type	RT1	RT2	RT3	RT4	CCS1	CCS2	CCS3	CCS4	meanCCS	stdCCS	RSD(%)
194	TG(56:6)>TG(18:1_18:1_20:4)_and_TG(16:0_18:1_22:5)_and_TG(18:1_18:2_20:3) + NH3	C59 H105 N O6	924.8013	TG	8.35	8.36	8.36	8.36	339.8	341.2	342.8	332.3	339.0	4.6	1.4
195	TG(56:4)>TG(18:1_18:2_20:1)_and_TG(18:1_18:1_20:2) + NH3	C59 H109 N O6	928.8322	TG	8.85	8.84	8.84	8.84	347.0	348.4	348.8	337.9	345.5	5.1	1.5
196	TG(57:2) + NH3	C60 H115 N O6	946.8795	TG	8.79	8.79	8.80	8.80	345.3	345.6	347.3	344.5	345.7	1.2	0.3
197	TG(58:8) + NH3	C61 H105 N O6	948.8010	TG	8.22	8.22	8.21	8.20	345.6	347.2	347.1	340.8	345.2	3.0	0.9
198	TG(57:1)>TG(16:0_18:1_23:0)_and_TG(18:0_18:1_21:0) + NH3	C60 H117 N O6	948.8952	TG	9.22	9.22	9.22	9.22	350.4	350.6	354.0	347.6	350.7	2.6	0.8
199	TG(58:4) + NH3	C61 H113 N O6	956.8637	TG	8.18	8.18	8.18	8.18	344.4	345.7	347.2	346.0	345.9	1.2	0.3
200	TG(58:3) + NH3	C61 H115 N O6	958.8795	TG	8.49	8.47	8.49	8.48	345.4	346.3	347.6	347.2	346.6	1.0	0.3
201	TG(58:2) + NH3	C61 H117 N O6	960.8951	TG	8.79	8.79	8.80	8.79	346.0	346.2	347.9	347.2	346.8	0.9	0.3
202	TG(58:1)>TG(16:0_18:1_24:0)_and_TG(16:0_16:0_26:1)_and_TG(18:0_18:1_22:0) + NH3	C61 H119 N O6	962.9108	TG	9.22	9.23	9.22	9.23	351.5	351.6	352.8	351.4	351.8	0.7	0.2

Supplementary Table S3. Summary of lipid features with matched ^{Orbi}CCS and ^{TIMS}CCS values from a single LC-Orbitrap experiment (pump speed 68%, resolution setting 240,000). The same dataset present in Fig. 3a-f.

#	Name	Type	m/z_this study	m/z_database	Error_ppm	RT (min)	Orbi_CCS	TIMS_CCS	% error
1	CE(20:4)M+NH3	CE	690.6186	690.6187	0.1	8.92	280.7	294	-4.5
2	CE(22:6)M+NH3	CE	714.6188	714.6185	0.7	8.57	287.3	295.9	-2.9
3	LPC(14:0)>LPC(14:0/0:0)_and_LPC(0:0/14:0)	LPC	468.3085	468.3084	0.6	8.7	214.2	224.6	-4.6
4	LPC(14:0)>LPC(14:0/0:0)_and_LPC(0:0/14:0)	LPC	468.3085	468.3087	0.1	1.67	214.5	225.5	-4.9
5	LPC(15:0)>LPC(15:0/0:0)_and_LPC(0:0/15:0)	LPC	482.3241	482.3245	0.4	1.57	220.1	223.3	-1.4
6	LPC(15:0)>LPC(15:0/0:0)_and_LPC(0:0/15:0)	LPC	482.3243	482.3243	0.4	1.82	220.7	228.3	-3.3
7	LPC(16:1)>LPC(16:1/0:0)_and_LPC(0:0/16:1)	LPC	494.3242	494.3244	1.2	2.03	222.1	228.5	-2.8
8	LPC(16:1)>LPC(16:1/0:0)_and_LPC(0:0/16:1)	LPC	494.3242	494.3243	0.5	2.77	221.7	227.1	-2.4
9	LPC(16:0)>LPC(16:0/0:0)_and_LPC(0:0/16:0)	LPC	496.3399	496.3402	0.2	1.69	220.4	233.7	-5.7
10	LPC(16:0)>LPC(16:0/0:0)_and_LPC(0:0/16:0)	LPC	496.3399	496.3405	0.3	1.78	220.7	232.1	-4.9
11	LPC(17:0)>LPC(17:0/0:0)_and_LPC(0:0/17:0)	LPC	510.3556	510.3555	0.4	2.22	225.4	236.4	-4.7
12	LPC(18:3)>LPC(18:3/0:0)_and_LPC(0:0/18:3)	LPC	518.3242	518.3242	0.1	2	230.0	226.1	1.7
13	LPC(20:5)>LPC(20:5/0:0)_and_LPC(0:0/20:5)	LPC	520.3397	520.3405	1.2	2.4	229.2	229.1	0.0
14	LPC(18:2)>LPC(18:2/0:0)_and_LPC(0:0/18:2)	LPC	520.3399	520.3402	0.4	2.77	229.6	231.2	-0.7
15	LPC(18:1)>LPC(18:1/0:0)_and_LPC(0:0/18:1)	LPC	522.3552	522.3562	1.1	2.78	229.2	235.2	-2.6
16	LPC(18:1)>LPC(18:1/0:0)_and_LPC(0:0/18:1)	LPC	522.3555	522.3554	0.2	2.17	229.4	236.9	-3.2
17	LPC(18:0)>LPC(18:0/0:0)_and_LPC(0:0/18:0)	LPC	524.3710	524.3717	1.5	1.91	229.8	240.1	-4.3
18	LPC(18:0)>LPC(18:0/0:0)_and_LPC(0:0/18:0)	LPC	524.3713	524.3715	0.7	1.84	229.9	241	-4.6
19	LPC(19:1)>LPC(19:1/0:0)_and_LPC(0:0/19:1)	LPC	536.3712	536.3720	0.0	1.69	237.6	237.8	-0.1
20	LPC(19:0)>LPC(19:0/0:0)_and_LPC(0:0/19:0)	LPC	538.3870	538.3872	1.1	1.65	239.8	243.2	-1.4
21	LPC(20:4)>LPC(20:4/0:0)_and_LPC(0:0/20:4)	LPC	544.3398	544.3399	0.4	2.54	235.8	232.6	1.4
22	LPC(20:4)>LPC(20:4/0:0)_and_LPC(0:0/20:4)	LPC	544.3398	544.3397	1.2	2.31	236.3	233.8	1.1
23	LPC(20:3)>LPC(20:3/0:0)_and_LPC(0:0/20:3)	LPC	546.3553	546.3552	0.1	2.74	235.9	233.8	0.9
24	LPC(20:3)>LPC(20:3/0:0)_and_LPC(0:0/20:3)	LPC	546.3559	546.3547	0.1	2.43	236.1	235.4	0.3
25	LPC(20:2)>LPC(20:2/0:0)_and_LPC(0:0/20:2)	LPC	548.3712	548.3708	0.8	2.23	238.7	236.5	0.9
26	LPC(20:1)>LPC(20:1/0:0)_and_LPC(0:0/20:1)	LPC	550.3868	550.3868	0.5	2.04	239.3	242.4	-1.3
27	LPC(20:0)>LPC(20:0/0:0)_and_LPC(0:0/20:0)	LPC	552.4027	552.4025	1.6	1.98	240.8	246.4	-2.3
28	LPC(22:6)>LPC(22:6/0:0)_and_LPC(0:0/22:6)	LPC	568.3398	568.3397	0.0	1.82	243.1	236.4	2.8
29	LPC(22:6)>LPC(22:6/0:0)_and_LPC(0:0/22:6)	LPC	568.3399	568.3396	0.1	1.89	243.3	235.2	3.5
30	LPC(22:5)>LPC(22:5/0:0)_and_LPC(0:0/22:5)	LPC	570.3555	570.3553	1.1	3.13	243.6	236.4	3.0
31	LPC(22:4)>LPC(22:4/0:0)_and_LPC(0:0/22:4)	LPC	572.3710	572.3710	0.1	2.16	245.7	238.6	3.0
32	LPC(22:0)>LPC(22:0/0:0)_and_LPC(0:0/22:0)	LPC	580.4339	580.4346	0.2	1.95	252.0	253.9	-0.7
33	PC(28:0)	PC	678.5071	678.5068	0.5	1.77	274.6	275.2	-0.2
34	PC(30:1)	PC	704.5226	704.5225	0.2	1.84	283.1	277.2	2.1
35	PC(30:0)	PC	706.5380	706.5382	0.3	3.53	280.0	280.5	-0.2
36	PC(O-32:2)	PC	716.5584	716.5588	0.3	4.82	284.5	282.4	0.7
37	PC(31:1)	PC	718.5385	718.5383	0.5	4.17	291.8	279.7	4.3
38	PC(O-32:1)	PC	718.5747	718.5748	2.6	4.88	283.5	285.2	-0.6
39	PC(O-32:0)	PC	720.5905	720.5898	3.3	4.33	284.3	287.9	-1.2
40	PC(32:3)	PC	728.5229	728.5231	1.0	4.05	285.4	277.1	3.0
41	PC(32:2)	PC	730.5385	730.5386	0.1	3.88	285.4	279.6	2.1
42	PC(32:1)	PC	732.5540	732.5543	2.5	5.69	285.3	283.3	0.7
43	PC(32:0)	PC	734.5697	734.5696	0.2	5.77	283.8	286.6	-1.0
44	PC(33:2)	PC	744.5543	744.5541	2.5	5.11	290.0	280.6	3.4
45	PC(O-34:2)	PC	744.5898	744.5890	0.5	4.58	289.7	287.6	0.7
46	PC(33:1)	PC	746.5696	746.5697	0.8	4.79	287.6	286.7	0.3
47	PC(O-34:1)	PC	746.6062	746.6054	2.1	4.54	290.8	291	-0.1
48	PC(O-34:0)	PC	748.6220	748.6205	0.2	4.21	293.0	294.4	-0.5

49	PC(34:4)	PC	754.5384	754.5381	0.4	3.67	292.5	281.9	3.8
50	PC(34:3)	PC	756.5540	756.5540	0.4	4.09	292.6	283.3	3.3
51	PC(34:2)	PC	758.5694	758.5718	0.2	3.59	290.0	286.4	1.3
52	PC(34:1)	PC	760.5852	760.5870	0.2	3.7	293.1	289.7	1.2
53	PC(34:0)	PC	762.6010	762.5990	0.4	3.36	297.8	292.8	1.7
54	PC(O-36:5)	PC	766.5751	766.5747	0.1	4.76	300.3	289.5	3.7
55	PC(35:4)	PC	768.5539	768.5540	0.0	4.01	297.6	284.3	4.7
56	PC(35:4)	PC	768.5539	768.5540	0.1	3.9	296.9	286.2	3.7
57	PC(O-36:4)	PC	768.5902	768.5895	0.4	3.62	294.8	291.7	1.1
58	PC(35:3)	PC	770.5694	770.5696	0.3	5.32	300.5	288	4.3
59	PC(O-36:3)	PC	770.6057	770.6059	0.7	4.58	296.5	291.8	1.6
60	PC(35:2)	PC	772.5851	772.5857	0.3	4.15	293.2	289.4	1.3
61	PC(O-36:2)	PC	772.6216	772.6210	0.3	3.9	300.0	294.3	1.9
62	PC(35:1)	PC	774.6008	774.6011	0.3	3.83	306.3	292.4	4.8
63	PC(O-36:1)	PC	774.6371	774.6368	1.3	6.22	301.8	297.4	1.5
64	PC(36:5)	PC	780.5540	780.5542	1.1	5.37	297.9	287.3	3.7
65	PC(O-37:5)	PC	780.5902	780.5903	0.1	4.47	299.4	290.7	3.0
66	PC(36:4)	PC	782.5694	782.5701	0.0	4.14	298.1	290.4	2.7
67	PC(36:4)	PC	782.5696	782.5695	0.8	6.51	297.3	287.5	3.4
68	PC(36:3)	PC	784.5851	784.5867	1.1	5.89	295.8	291.1	1.6
69	PC(36:2)	PC	786.6010	786.6026	0.0	4.26	297.1	292.7	1.5
70	PC(36:1)	PC	788.6166	788.6167	0.3	4.05	300.1	295.4	1.6
71	PC(O-38:5)	PC	794.6051	794.6055	0.7	3.79	302.3	295.7	2.2
72	PC(O-38:4)	PC	796.6217	796.6210	0.5	5.44	306.7	297.5	3.1
73	PC(37:2)	PC	800.6160	800.6174	0.3	4.42	301.5	295.5	2.0
74	PC(37:1)	PC	802.6323	802.6332	0.3	5.9	306.4	298.3	2.7
75	PC(36:3)	PC	806.5695	806.5692	0.7	5.32	305.6	293.1	4.3
76	PC(38:6)	PC	806.5695	806.5689	0.1	4.2	305.7	291.4	4.9
77	PC(38:5)	PC	808.5850	808.5848	1.4	6.26	301.8	294.2	2.6
78	PC(38:4)	PC	810.6008	810.6011	0.0	5.83	304.7	296.7	2.7
79	PC(38:3)	PC	812.6165	812.6163	0.8	5.38	307.1	297	3.4
80	PC(38:2)	PC	814.6321	814.6311	0.0	4.66	307.9	298.4	3.2
81	PC(38:1)	PC	816.6475	816.6468	0.2	4.53	307.7	300.8	2.3
82	PC(O-40:7)	PC	818.6057	818.6057	1.9	6.3	313.2	298	5.1
83	PC(39:6)	PC	820.5850	820.5850	1.3	5.46	308.1	296.6	3.9
84	PC(O-40:6)	PC	820.6211	820.6209	1.6	5.32	315.0	298.9	5.4
85	PC(39:4)	PC	824.6166	824.6160	1.0	6.36	310.1	299.7	3.5
86	PC(O-40:4)	PC	824.6523	824.6529	0.9	5.7	321.5	302.8	6.2
87	PC(40:8)	PC	830.5696	830.5690	0.1	5.03	312.5	294	6.3
88	PC(40:7)	PC	832.5848	832.5846	1.0	4.7	312.6	296.8	5.3
89	PC(40:6)	PC	834.6007	834.6002	0.4	4.21	312.0	299.5	4.2
90	PC(42:7)	PC	838.6320	838.6315	0.0	6.47	314.8	301.8	4.3
91	PC(O-42:6)	PC	848.6526	848.6528	0.9	5.57	319.9	304.2	5.1
92	PC(42:9)	PC	856.5850	856.5846	0.4	5.11	321.5	297.6	8.0
93	PC(42:5)	PC	864.6475	864.6473	0.7	6.44	325.8	305.5	6.7
94	PC(42:4)	PC	866.6638	866.6643	0.3	5.35	325.0	308.8	5.3
95	PE(40:5)	PE	794.5693	794.5694	0.1	4.53	303.4	291.1	4.2
96	SM(d32:2)	SM	673.5282	673.5277	0.2	6.24	271.6	276.9	-1.9
97	SM(d32:1)	SM	675.5438	675.5447	0.1	3.92	271.5	280.5	-3.2

98	SM(d32:0)	SM	677.5598	677.5582	2.0	5.2	278.2	285	-2.4
99	SM(d33:1)	SM	689.5595	689.5599	0.5	3.04	277.3	283.8	-2.3
100	SM(d34:2)	SM	701.5595	701.5615	4.2	4.06	278.1	283.5	-1.9
101	SM(d34:1)	SM	703.5750	703.5778	3.0	3.58	277.6	286.3	-3.0
102	SM(d34:0)	SM	705.5904	705.5891	0.7	4.81	281.2	290.1	-3.1
103	SM(d35:2)	SM	715.5750	715.5748	0.1	4.19	286.7	286.6	0.0
104	SM(d35:1)	SM	717.5906	717.5905	2.1	6.46	283.3	289.8	-2.2
105	SM(d34:0-OH)	SM	721.5858	721.5842	0.2	3.24	287.1	290.4	-1.1
106	SM(d36:3)	SM	727.5753	727.5748	1.9	3.7	285.3	286.8	-0.5
107	SM(d36:2)	SM	729.5908	729.5904	1.4	3.5	285.7	289.7	-1.4
108	SM(d36:1)	SM	731.6065	731.6062	0.7	3.1	284.2	292.6	-2.9
109	SM(d36:0)	SM	733.6223	733.6208	0.8	3.76	293.3	295.7	-0.8
110	SM(d37:1)	SM	745.6221	745.6221	1.6	4.33	293.9	295.2	-0.4
111	SM(d38:2)	SM	757.6218	757.6216	0.2	4.43	291.0	295.6	-1.6
112	SM(d38:1)	SM	759.6378	759.6373	0.3	3.83	292.9	298.2	-1.8
113	SM(d39:1)	SM	773.6533	773.6531	1.3	5.11	296.5	300.8	-1.4
114	SM(d40:2)	SM	785.6533	785.6531	0.7	3.72	301.7	301.2	0.2
115	SM(d40:1)	SM	787.6690	787.6697	0.2	5.29	302.4	303	-0.2
116	SM(d41:2)	SM	799.6690	799.6687	0.3	5.77	303.7	302.8	0.3
117	SM(d41:1)	SM	801.6846	801.6854	0.2	6.23	304.1	306.5	-0.8
118	SM(d42:3)	SM	811.6686	811.6685	1.1	6.54	308.7	303.9	1.6
119	SM(d42:2)	SM	813.6845	813.6862	0.2	5.91	309.2	306.6	0.8
120	SM(d43:3)	SM	825.6846	825.6846	1.1	6.75	311.9	306	1.9
121	SM(d43:2)	SM	827.7000	827.6999	0.3	6.1	313.8	310.1	1.2
122	SM(d44:3)	SM	839.6996	839.6997	0.2	5.8	315.6	310.6	1.6
123	TG(44:1)>TG(10:0_16:0_18:1) M + NH3	TG	766.6920	766.6927	0.1	6.62	296.3	299.8	-1.2
124	TG(46:2) M + NH3	TG	792.7078	792.7083	0.1	6.24	302.3	303.3	-0.3
125	TG(46:1)>TG(12:0_16:0_18:1)_and_TG(10:0_18:0_18:1)_and_TG(14:0_16:0_16:1) M + NH3	TG	794.7234	794.7233	0.1	6.56	302.1	305.5	-1.1
126	TG(46:0)>TG(14:0_16:0_16:0)_and_TG(12:0_16:0_18:0) M + NH3	TG	796.7388	796.7391	0.8	7.76	309.5	307.8	0.5
127	TG(48:3) M + NH3	TG	818.7234	818.7238	0.7	7.79	309.7	307.4	0.7
128	TG(48:2)>TG(14:0_16:0_18:2)_and_TG(14:0_16:1_18:1) M + NH3	TG	820.7391	820.7395	0.1	8.44	309.3	309.3	0.0
129	TG(48:1)>TG(16:0_16:0_16:1)_and_TG(14:0_16:0_18:1) M + NH3	TG	822.7548	822.7547	0.1	8.07	309.4	311.6	-0.7
130	TG(49:3) M + NH3	TG	832.7389	832.7390	0.1	8.42	315.1	310.9	1.3
131	TG(49:2) M + NH3	TG	834.7545	834.7550	0.5	8.1	315.2	312.2	1.0
132	TG(49:1)>TG(15:0_16:0_18:0)_and_TG(16:0_16:0_17:1)_and_TG(16:0_16:1_17:0) M + NH3	TG	836.7702	836.7703	0.9	7.86	319.5	314.6	1.6
133	TG(50:5) M + NH3	TG	842.7190	842.7231	0.2	8.61	315.7	310.8	1.6
134	TG(50:4)>TG(16:1_16:1_18:2)_and_TG(16:1_16:1_18:2) M + NH3	TG	844.7388	844.7396	0.1	7.96	315.4	311.6	1.2
135	TG(50:3) M + NH3	TG	846.7546	846.7556	0.1	8.01	316.2	313.5	0.9
136	TG(50:2)>TG(16:0_16:1_18:1)_and_TG(14:0_18:1_18:1) M + NH3	TG	848.7701	848.7708	0.9	7.75	317.4	315.3	0.7
137	TG(50:1)>TG(16:0_16:0_18:1)_M+NH3	TG	850.7858	850.7857	0.3	7.75	316.8	318	-0.4
138	TG(51:4)>TG(16:1_17:1_18:2)_and_TG(15:0_18:2_18:2)_and_TG(15:1_18:1_18:2) M + NH3	TG	858.7545	858.7544	0.1	8.81	319.8	314.9	1.5

139	TG(51:2)>TG(15:0_18:1_18:1)_and_TG(16:0_17:1_18:1)_and_TG(16:1_17:0_18:1) M + NH3	TG	862.7857	862.7861	0.8	8.42	320.2	318.2	0.6
140	TG(51:1) M + NH3	TG	864.8014	864.8019	1.3	9.22	321.1	320.8	0.1
141	TG(52:6) M + NH3	TG	868.7388	868.7387	0.8	8.42	319.4	314.8	1.5
142	TG(52:5)>TG(16:0_18:2_18:3) M + NH3	TG	870.7545	870.7554	0.1	7.79	320.4	316.2	1.3
143	TG(52:4)>TG(16:0_16:0_20:4)_and_TG(16:1_18:1_18:2)_and_TG(16:0_18:1_18:2) M + NH3	TG	872.7701	872.7720	0.7	9.22	320.8	317.7	1.0
144	TG(52:3)>TG(16:0_18:1_18:2)_and_TG(16:1_18:1_18:1) M + NH3	TG	874.7858	874.7875	1.0	8.79	321.5	320	0.5
145	TG(52:2)>TG(16:0_18:1_18:1) M + NH3	TG	876.8016	876.8023	2.0	8.48	337.1	321.9	4.7
146	TG(52:1) M + NH3	TG	878.8170	878.8164	2.0	8.18	324.3	324	0.1
147	TG(53:5)>TG(17:1_18:2_18:2) M + NH3	TG	884.7701	884.7688	0.8	7.85	329.6	319.5	3.2
148	TG(53:4)>TG(17:0_18:2_18:2)_and_TG(17:1_18:1_18:2) M + NH3	TG	886.7856	886.7852	1.2	8.12	329.7	321.1	2.7
149	TG(53:3)>TG(17:0_18:1_18:2)_and_TG(17:1_18:1_18:1)_and_TG(16:1_18:1_19:1)_and_TG(16:0_18:1_19:2) M + NH3	TG	888.8013	888.8012	0.5	7.81	330.1	323.1	2.2
150	TG(53:2) M + NH3	TG	890.8167	890.8168	1.0	7.94	334.1	324.9	2.8
151	TG(54:7)>TG(16:0_16:1_22:6)_and_TG(16:0_18:2_20:5) M + NH3	TG	894.7543	894.7539	0.1	7.89	328.1	318.5	3.0
152	TG(52:3)>TG(16:0_18:1_18:2)_and_TG(16:1_18:1_18:1) M + NH3	TG	896.7701	896.7699	0.2	9	329.5	320.4	2.9
153	TG(54:5)>TG(18:1_18:2_18:2)_and_TG(16:0_16:0_22:5)_and_TG(16:0_18:1_20:4) M + NH3	TG	898.7858	898.7855	0.0	8.59	329.9	322	2.5
154	TG(54:4)>TG(18:1_18:1_18:2) M + NH3	TG	900.8012	900.8010	0.6	8.3	331.5	323.8	2.4
155	TG(54:3)>TG(18:1/18:1/18:1) M + NH3	TG	902.8167	902.8167	1.4	8.02	332.1	325.8	1.9
156	TG(54:2)>TG(16:0_18:1_20:1)_and_TG(18:0_18:1_18:1) M + NH3	TG	904.8326	904.8316	0.0	7.91	335.1	327.7	2.3
157	TG(56:6)>TG(18:1_18:1_20:4)_and_TG(16:0_18:1_22:5)_and_TG(18:1_18:2_20:3) M + NH3	TG	924.8013	924.8006	1.1	9.2	341.2	327.6	4.2
158	TG(56:5)>TG(16:0_18:1_22:4) M + NH3	TG	926.8172	926.8162	0.2	8.87	344.6	329.2	4.7
159	TG(56:1) M + NH3	TG	934.8792	934.8793	0.1	8.47	346.9	335.2	3.5
160	TG(58:10) M + NH3	TG	944.7701	944.7694	0.4	8.12	343.6	326.7	5.2
161	TG(57:2) M + NH3	TG	946.8793	946.8787	0.6	8.36	345.6	335.8	2.9
162	TG(57:1)>TG(16:0_18:1_23:0)_and_TG(18:0_18:1_21:0) M + NH3	TG	948.8951	948.8944	1.0	8.8	350.6	337.2	4.0
163	TG(58:3) M + NH3	TG	958.8792	958.8787	0.6	8.79	346.3	336.4	2.9
164	TG(58:2) M + NH3	TG	960.8950	960.8946	0.2	8.36	346.2	338.4	2.3

Supplementary Table S4. Summary of lipid features with matched ^{Orbi}CCS and ^{DT}CCS values from a single LC-Orbitrap experiment (pump speed 68%, resolution setting 240,000). The same dataset present in Fig. 3a-f.

#	Name	Type	m/z_this study	m/z_database	mzError_ppm	RT (min)	Orbi_CCS	DT_CCS	% error
1	LPC(16:0)>LPC(16:0/0:0)_and_LPC(0:0/16:0)	LPC	496.33994	496.3403	0.7	2.77	218.2	231.4	-5.7
2	LPC(18:0)>LPC(18:0/0:0)_and_LPC(0:0/18:0)	LPC	524.37122	524.3716	0.7	2.77	228.4	238.8	-4.3
3	LPC(20:0)>LPC(20:0/0:0)_and_LPC(0:0/20:0)	LPC	552.40259	552.4029	0.6	2.74	242.6	247	-1.8
4	Car(12:0)	Car	344.27948	344.2782	3.7	1.23	172.1	199.5	-13.7
5	Car(16:0)	Car	400.3421	400.3427	1.5	2.05	187.2	214.7	-12.8
6	LPC(14:0)>LPC(14:0/0:0)_and_LPC(0:0/14:0)	LPC	468.3085	468.309	1.1	1.57	212.5	220.8	-3.8
7	LPC(18:1)>LPC(18:1/0:0)_and_LPC(0:0/18:1)	LPC	522.35547	522.3559	0.8	2.17	229.3	233.2	-1.7
8	LPC(19:0)>LPC(19:0/0:0)_and_LPC(0:0/19:0)	LPC	538.38696	538.3872	0.4	2.54	240.0	240.5	-0.2
9	PC(32:2)	PC	730.53845	730.5387	0.3	3.70	283.4	278.4	1.8
10	PC(34:1)	PC	760.58502	760.5856	0.8	4.88	292.9	287.9	1.7
11	PC(36:2)	PC	786.60065	786.6012	0.7	5.11	294.2	293.3	0.3
12	PC(32:1)	PC	732.55389	732.5543	0.6	4.17	283.4	277.6	2.1
13	PC(34:2)	PC	758.56927	758.5699	0.8	4.33	290.7	280.6	3.6
14	PC(34:3)	PC	756.55396	756.5543	0.4	3.90	290.2	278.2	4.3
15	PC(35:1)	PC	774.60089	774.6013	0.5	5.32	292.8	285.8	2.5
16	PC(36:1)	PC	788.61652	788.6169	0.5	5.77	294.3	289.4	1.7
17	PC(36:2)	PC	786.60065	786.6012	0.7	4.96	294.2	287.1	2.5
18	PE(O-36:3)>PE(O-18:1/18:2)	PE	728.55927	728.5594	0.2	5.59	286.5	273.5	4.8
19	SM(d34:1)	SM	703.57489	703.5754	0.7	4.06	276.6	281.2	-1.6
20	SM(d36:1)	SM	731.60669	731.6067	0.0	4.81	284.1	288.4	-1.5
21	SM(d36:2)	SM	729.59052	729.591	0.7	4.19	285.4	285.3	0.1
22	SM(d37:1)	SM	745.62195	745.6223	0.5	5.29	290.8	289.8	0.4
23	SM(d38:1)	SM	759.63751	759.638	0.6	5.77	292.3	293.4	-0.4
24	SM(d39:1)	SM	773.65338	773.6536	0.3	5.97	294.9	297	-0.7
25	SM(d40:1)	SM	787.66882	787.6693	0.6	6.54	298.5	299.1	-0.2
26	SM(d40:2)	SM	785.65332	785.6536	0.4	5.91	298.5	296.8	0.6
27	SM(d41:1)	SM	801.68457	801.6849	0.4	6.75	300.4	302.3	-0.6
28	SM(d41:2)	SM	799.66901	799.6693	0.4	6.34	301.1	300.1	0.3
29	SM(d42:2)	SM	813.68445	813.6849	0.6	6.46	304.5	302.2	0.8
30	SM(d42:3)	SM	811.6687	811.6693	0.7	5.80	304.6	300.8	1.3
31	SM(d43:2)	SM	827.70013	827.7006	0.6	6.81	307.8	305.7	0.7

Supplementary Table S5. Isotopically labeled lipid standards. Lipid names, CAS numbers, and concentrations of isotopically labeled standards purchased from Avanti Polar Lipids (Alabaster, AL, USA). The internal standard stock solution was prepared in-house and used for quantification and normalization in the reverse-phase UHPLC–MS workflow.

Lipid Subclass Name	Isotopically Labeled Lipids	CAS Number	Concentration (μg mL^{-1})
Cholesterol Ester	CE (18:1(d7))	1416275-35-7	350
Cholesterol	Cholesterol-d7	83199-47-7	100
Diacylglycerol	DG (15:0/18:1(d7))	2097561-14-1	10
Lysophosphatidylcholine	LPC (18:1(d7))	2097561-13-0	25
Lysophosphatidylethanolamine	LPE (18:1(d7))	2260669-47-2	5
Phosphatidylcholine	PC (15:0/18:1(d7))	2097561-16-3	160
Phosphatidylethanolamine	PE (15:0/18:1(d7))	2097561-15-2	5
Phosphatidylglycerol	PG (15:0/18:1(d7))	2260669-42-7	30
Phosphatidylinositol	PI (15:0/18:1(d7))	2260669-44-9	20
Phosphatidylserine	PS (15:0/18:1(d7))	2260669-40-5	10
Sphingomyelin	SM (d18:1/(d9))	2260669-50-7	30
Triacylglycerol	TG (15:0/18:1(d7)/15:0)	2097561-17-4	55

Supplementary Figure S6. Slope and intercept values used to calibrate ^{Orbi}CCS during an LC experiment. Panel (a) shows the changing ion gauge pressure reading when the experiment was performed at reduced turbopump speed. Panel (b) and (c) show the corresponding RT-specific calibration factors, a_{RT} and b_{RT} from linear regression analysis between ^{DT}CCS values of internal standards and their corresponding $^{Orbi}CCS_{LC,IS}$.

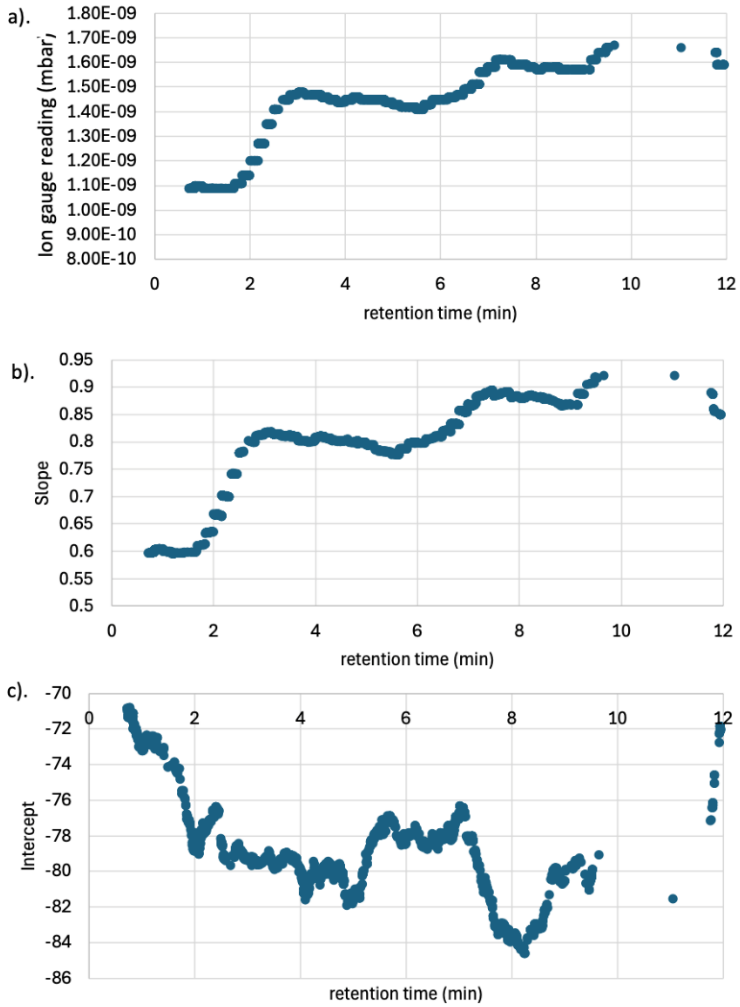

Supplementary Table S7. Summary of lipid features with matched ^{Orbi}CCS to reference ^{TIMS}CCS dataset for experiments conducted at a resolution of 240,000 and turbopump speed 68%.

#	m/z_this study	m/z_database	mzError_ppm	^{Orbi}CCS_this study	^{TIMS}CCS_database	Name_database	Name_This study	% error
1	818.7234	818.7238	0.5	307.4	307.4	TG 16:1_16:1_16:1	TG(48:3) M + NH3	0.0
2	796.7390	796.7391	0.1	307.9	307.8	TG 14:0_16:0_16:0	TG(46:0)>TG(14:0_16:0_16:0)_and_TG(12:0_16:0_18:0) M + NH3	0.0
3	580.4340	580.4346	1.1	254.0	253.9	LPC 22:0-SN1	LPC(22:0)>LPC(22:0/0:0)_and_LPC(0:0/22:0)	0.0
4	846.7546	846.7556	1.2	313.4	313.5	TG 16:1_16:0_18:2	TG(50:3) M + NH3	0.0
5	716.5590	716.5588	0.2	282.3	282.4	PC O-32:2	PC(O-32:2)	0.0
6	732.5539	732.5543	0.5	283.4	283.3	PC 16:0_16:1	PC(32:1)	0.1
7	872.7703	872.7720	2.0	317.9	317.7	TG 16:0_18:2_18:2	TG(52:4)>TG(16:0_16:0_20:4)_and_TG(16:1_18:1_18:2)_and_TG(16:0_18:1_18:2) M + NH3	0.1
8	746.5698	746.5697	0.1	287.0	286.7	PC 33:1	PC(33:1)	0.1
9	796.6216	796.6210	0.9	297.8	297.5	PC O-38:4	PC(O-38:4)	0.1
10	820.7391	820.7395	0.5	309.6	309.3	TG 14:0_16:1_18:1	TG(48:2)>TG(14:0_16:0_18:2)_and_TG(14:0_16:1_18:1) M + NH3	0.1
11	794.7234	794.7233	0.1	305.2	305.5	TG 14:0_16:0_16:1	TG(46:1)>TG(12:0_16:0_18:1)_and_TG(10:0_18:0_18:1)_and_TG(14:0_16:0_16:1) M + NH3	-0.1
12	774.6009	774.6011	0.3	292.8	292.4	PC 35:1	PC(35:1)	0.2
13	792.7077	792.7083	0.7	302.8	303.3	TG 12:0_16:1_18:1	TG(46:2) M + NH3	-0.2
14	848.7701	848.7708	0.8	314.7	315.3	TG 16:0_16:1_18:1	TG(50:2)>TG(16:0_16:1_18:1)_and_TG(14:0_18:1_18:1) M + NH3	-0.2
15	770.6058	770.6059	0.1	292.4	291.8	PC O-36:3	PC(O-36:3)	0.2
16	900.8010	900.8010	0.1	324.4	323.8	TG 18:1_18:1_18:2	TG(54:4)>TG(18:1_18:1_18:2) M + NH3	0.2
17	520.3397	520.3405	1.5	229.6	229.1	LPC 18:2-SN1	LPC(18:2)>LPC(18:2/0:0)_and_LPC(0:0/18:2)	0.2
18	811.6687	811.6685	0.2	304.6	303.9	SM d42:3	SM(d42:3)	0.2
19	744.5902	744.5890	1.6	288.3	287.6	PC O-34:2	PC(O-34:2)	0.2
20	844.7388	844.7396	0.9	312.4	311.6	TG 14:0_18:2_18:2	TG(50:4)>TG(16:1_16:1_18:2)_and_TG(16:1_16:1_18:2) M + NH3	0.3

21	546.3556	546.3547	1.6	236.2	235.4	LPC 20:3-SN2	LPC(20:3)>LPC(20:3/0:0)_and_LPC(0:0/20:3)	0.3
22	768.5903	768.5895	1.0	290.7	291.7	PC O-36:4	PC(O-36:4)	-0.3
23	902.8168	902.8167	0.2	324.7	325.8	TG 18:1_18:1_18:1	TG(54:3)>TG(18:0_18:1_18:2)_and_TG(18:1/18:1/18:1)_and_TG(16:0_18:2_20:1) M + NH3	-0.4
24	788.6165	788.6167	0.2	294.3	295.4	PC 18:0_18:1	PC(36:1)	-0.4
25	870.7545	870.7554	1.0	317.4	316.2	TG 16:1_18:2_18:2	TG(52:5)>TG(16:0_18:2_18:3) M + NH3	0.4
26	850.7858	850.7857	0.1	316.8	318	TG 16:0_16:0_18:1	TG(50:1)>TG(16:0_16:0_18:1)_M+NH3	-0.4
27	678.5070	678.5068	0.3	276.3	275.2	PC 14:0_14:0	PC(28:0)	0.4
28	727.5753	727.5748	0.7	285.7	286.8	SM d36:3	SM(d36:3)	-0.4
29	550.3868	550.3868	0.1	241.4	242.4	LPC 20:1-SN1	LPC(20:1)>LPC(20:1/0:0)_and_LPC(0:0/20:1)	-0.4
30	830.7236	830.7228	0.9	308.0	309.4	TG 15:1_16:1_18:2	TG(49:4) M + NH3	-0.4
31	799.6690	799.6687	0.3	301.4	302.8	SM d41:2	SM(d41:2)	-0.4
32	862.7860	862.7861	0.1	319.6	318.2	TG 16:0_17:1_18:1	TG(51:2) M + NH3	0.5
33	520.3398	520.3402	0.7	230.1	231.2	LPC 18:2-SN2	LPC(18:2)>LPC(18:2/0:0)_and_LPC(0:0/18:2)	-0.5
34	874.7858	874.7875	2.0	318.3	320	TG 16:0_18:1_18:2	TG(52:3)>TG(16:0_18:1_18:2)_and_TG(16:1_18:1_18:1) M + NH3	-0.5
35	786.6007	786.6026	2.5	294.2	292.7	PC 18:0_18:2	PC(36:2)	0.5
36	536.3713	536.3720	1.2	239.0	237.8	LPC 19:1-SN1	LPC(19:1)>LPC(19:1/0:0)_and_LPC(0:0/19:1)	0.5
37	794.6052	794.6055	0.4	297.3	295.7	PC O-38:5	PC(O-38:5)	0.5
38	827.7000	827.6999	0.1	308.4	310.1	SM d43:2	SM(d43:2)	-0.6
39	822.7546	822.7547	0.1	309.9	311.6	TG 14:0_16:0_18:1	TG(48:1)>TG(16:0_16:0_16:1)_and_TG(14:0_16:0_18:1) M + NH3	-0.6
40	888.8013	888.8012	0.0	324.9	323.1	TG 17:0_18:1_18:2	TG(53:3)>TG(17:0_18:1_18:2)_and_TG(17:1_18:1_18:1)_and_TG(16:1_18:1_19:1)_and_TG(16:0_18:1_19:2) M + NH3	0.6
41	839.6998	839.6997	0.1	312.4	310.6	SM d44:3	SM(d44:3)	0.6
42	898.7857	898.7855	0.2	324.0	322	TG 18:1_18:2_18:2	TG(54:5)>TG(18:1_18:2_18:2)_and_TG(16:0_16:0_22:5)_and_TG(16:0_18:1_20:4) M + NH3	0.6

43	878.8170	878.8164	0.7	321.8	324	TG 16:0_18:0_18:1	TG(52:1) M + NH3	-0.7
44	813.6845	813.6862	2.1	304.5	306.6	SM d18:1_24:1	SM(d42:2)	-0.7
45	868.7387	868.7387	0.1	317.0	314.8	TG 16:1_18:2_18:3	TG(52:6) M + NH3	0.7
46	762.6009	762.5990	2.5	290.6	292.8	PC 18:0_16:0	PC(34:0)	-0.8
47	825.6845	825.6846	0.1	308.3	306	SM d43:3	SM(d43:3)	0.8
48	544.3397	544.3397	0.1	235.6	233.8	LPC 20:4-SN2	LPC(20:4)>LPC(20:4/0:0)_and_LPC(0:0/20:4)	0.8
49	824.7708	824.7706	0.3	311.3	313.8	TG 16:0_16:0_16:0	TG(48:0)>TG(16:0/16:0/16:0)_and_TG(14:0_16:0_18:0) M + NH3	-0.8
50	546.3555	546.3552	0.5	235.7	233.8	LPC 20:3-SN1	LPC(20:3)>LPC(20:3/0:0)_and_LPC(0:0/20:3)	0.8
51	824.6523	824.6529	0.7	305.3	302.8	PC O-40:4	PC(O-40:4)	0.8
52	706.5379	706.5382	0.4	278.1	280.5	PC 30:0	PC(30:0)	-0.8
53	864.8012	864.8019	0.8	323.6	320.8	TG 16:0_17:0_18:1	TG(51:1) M + NH3	0.9
54	784.5850	784.5867	2.1	293.6	291.1	PC 18:1_18:2	PC(36:3)	0.9
55	876.8015	876.8023	1.0	319.1	321.9	TG 16:0_18:1_18:1	TG(52:2)>TG(16:0_18:1_18:1) M + NH3	-0.9
56	858.7545	858.7544	0.1	317.7	314.9	TG 15:0_18:2_18:2	TG(51:4)>TG(16:1_17:1_18:2)_and_TG(15:0_18:2_18:2)_and_TG(15:1_18:1_18:2) M + NH3	0.9
57	832.7390	832.7390	0.1	308.1	310.9	TG 15:0_16:1_18:2	TG(49:3) M + NH3	-0.9
58	785.6533	785.6531	0.2	298.5	301.2	SM d40:2	SM(d40:2)	-0.9
59	548.3712	548.3708	0.8	238.8	236.5	LPC 20:2-SN1	LPC(20:2)>LPC(20:2/0:0)_and_LPC(0:0/20:2)	1.0
60	772.6217	772.6210	0.9	291.4	294.3	PC O-36:2	PC(O-36:2)	-1.0
61	766.6921	766.6927	0.8	296.8	299.8	TG 12:0_16:0_16:1	TG(44:1)>TG(10:0_16:0_18:1) M + NH3	-1.0
62	896.7700	896.7699	0.0	323.6	320.4	TG 18:2_18:2_18:2	TG(54:6)>TG(16:0_18:2_20:4) M + NH3	1.0
63	718.5748	718.5748	0.0	282.3	285.2	PC O-32:1	PC(O-32:1)	-1.0
64	836.7701	836.7703	0.2	311.3	314.6	TG 15:0_16:0_18:1	TG(49:1)>TG(15:0_16:0_18:0)_and_TG(16:0_16:0_17:1)_and_TG(16:0_16:1_17:0) M + NH3	-1.0

65	746.6063	746.6054	1.3	287.9	291	PC O-34:1	PC(O-34:1)	-1.1
66	715.5750	715.5748	0.3	283.5	286.6	SM d35:2	SM(d35:2)	-1.1
67	904.8325	904.8316	1.1	331.2	327.7	TG 18:0_18:1_18:1	TG(54:2)>TG(16:0_18:1_20:1)_and_TG(18:0_18:1_18:1) M + NH3	1.1
68	772.5852	772.5857	0.7	292.5	289.4	PC 35:2	PC(35:2)	1.1
69	886.7857	886.7852	0.6	324.6	321.1	TG 17:1_18:1_18:2	TG(53:4)>TG(17:0_18:2_18:2)_and_TG(17:1_18:1_18:2) M + NH3	1.1
70	760.5850	760.5870	2.6	292.9	289.7	PC 16:0_18:1	PC(34:1)	1.1
71	734.5698	734.5696	0.3	283.0	286.6	PC 16:0_16:0	PC(32:0)	-1.3
72	810.6007	810.6011	0.5	300.4	296.7	PC 18:0_20:4	PC(38:4)	1.3
73	518.3242	518.3248	1.1	230.8	227.9	LPC 18:3-SN2	LPC(18:3)>LPC(18:3/0:0)_and_LPC(0:0/18:3)	1.3
74	882.7543	882.7543	0.0	322.5	318.4	TG 17:1_18:2_18:3	TG(53:6)>TG(17:2_18:2_18:2)_and_TG(17:1_18:2_18:3) M + NH3	1.3
75	538.3870	538.3872	0.4	240.0	243.2	LPC 19:0-SN2	LPC(19:0)>LPC(19:0/0:0)_and_LPC(0:0/19:0)	-1.3
76	796.5848	796.5848	0.1	297.7	293.8	PC 37:4	PC(37:4)	1.3
77	730.5385	730.5386	0.2	283.4	279.6	PC 32:2	PC(32:2)	1.3
78	782.5693	782.5701	1.0	294.4	290.4	PC 16:0_20:4	PC(36:4)	1.4
79	721.5856	721.5842	2.0	286.3	290.4	SM 34:0;3O	SM(d34:0-OH)	-1.4
80	924.8012	924.8006	0.6	332.3	327.6	TG 18:1_18:1_20:4	TG(56:6)>TG(18:1_18:1_20:4)_and_TG(16:0_18:1_22:5)_and_TG(18:1_18:2_20:3) M + NH3	1.4
81	884.7700	884.7688	1.4	324.1	319.5	TG 17:1_18:2_18:2	TG(53:5)>TG(17:1_18:2_18:2) M + NH3	1.4
82	729.5905	729.5904	0.1	285.4	289.7	SM d18:1_18:1	SM(d36:2)	-1.5
83	787.6688	787.6697	1.1	298.5	303	SM d40:1	SM(d40:1)	-1.5
84	814.6320	814.6311	1.1	302.8	298.4	PC 38:2	PC(38:2)	1.5
85	745.6220	745.6221	0.2	290.8	295.2	SM d37:1	SM(d37:1)	-1.5
86	758.5693	758.5718	3.3	290.7	286.4	PC 16:0_18:2	PC(34:2)	1.5
87	544.3398	544.3399	0.0	236.1	232.6	LPC 20:4-SN1	LPC(20:4)>LPC(20:4/0:0)_and_LPC(0:0/20:4)	1.5

88	552.4026	552.4025	0.1	242.6	246.4	LPC 20:0-SN1	LPC(20:0)>LPC(20:0/0:0)_and_LPC(0:0/20:0)	-1.6
89	802.6322	802.6332	1.3	303.1	298.3	PC 37:1	PC(37:1)	1.6
90	714.6189	714.6185	0.6	291.1	295.9	CE 22:6	CE(22:6)M+NH3	-1.6
91	688.6030	688.6026	0.7	296.3	291.4	CE 20:5	CE(20:5)M+NH3	1.7
92	704.5224	704.5225	0.2	282.0	277.2	PC 30:1	PC(30:1)	1.7
93	800.6165	800.6174	1.1	300.7	295.5	PC 37:2	PC(37:2)	1.8
94	673.5281	673.5277	0.7	271.8	276.9	SM d32:2	SM(d32:2)	-1.8
95	744.5540	744.5540	0.0	288.1	282.7	PC 33:2	PC(33:2)	1.9
96	748.6220	748.6205	1.9	288.7	294.4	PC O-34:0	PC(O-34:0)	-1.9
97	733.6217	733.6208	1.3	289.9	295.7	SM d36:0	SM(d36:0)	-2.0
98	801.6846	801.6854	1.1	300.4	306.5	SM d41:1	SM(d41:1)	-2.0
99	759.6375	759.6373	0.3	292.3	298.2	SM d38:1	SM(d38:1)	-2.0
100	718.5383	718.5383	0.1	285.3	279.7	PC 17:0_14:1	PC(31:1)	2.0
101	518.3242	518.3242	0.0	230.6	226.1	LPC 18:3-SN1	LPC(18:3)>LPC(18:3/0:0)_and_LPC(0:0/18:3)	2.0
102	838.6318	838.6315	0.3	307.9	301.8	PC 40:4	PC(40:4)	2.0
103	773.6533	773.6531	0.2	294.7	300.8	SM d39:1	SM(d39:1)	-2.0
104	816.6475	816.6468	0.8	306.9	300.8	PC 38:1	PC(38:1)	2.0
105	820.6212	820.6209	0.3	305.1	298.9	PC O-40:6	PC(O-40:6)	2.1
106	720.5904	720.5898	0.8	281.7	287.9	PC O-32:0	PC(O-32:0)	-2.1
107	717.5906	717.5905	0.2	283.5	289.8	SM d35:1	SM(d35:1)	-2.2
108	890.8170	890.8168	0.2	331.9	324.9	TG 17:0_18:1_18:1	TG(53:2) M + NH3	2.2
109	728.5228	728.5231	0.4	283.2	277.1	PC 32:3	PC(32:3)	2.2
110	494.3242	494.3243	0.2	222.1	227.1	LPC 16:1-SN1	LPC(16:1)>LPC(16:1/0:0)_and_LPC(0:0/16:1)	-2.2
111	818.6056	818.6057	0.1	304.7	298	PC O-40:7	PC(O-40:7)	2.3

112	834.6008	834.6002	0.8	306.4	299.5	PC 18:0_22:6	PC(40:6)	2.3
113	770.5694	770.5696	0.3	294.7	288	PC 35:3	PC(35:3)	2.3
114	701.5594	701.5615	3.0	276.9	283.5	SM d18:1_16:1	SM(d34:2)	-2.3
115	661.5283	661.5282	0.2	271.1	277.7	SM d31:1	SM(d31:1)	-2.4
116	768.5538	768.5540	0.3	293.0	286.2	PC 35:4	PC(35:4)	2.4
117	782.5693	782.5695	0.2	294.4	287.5	PC 18:2_18:2 Cis	PC(36:4)	2.4
118	808.5848	808.5848	0.0	301.4	294.2	PC 38:5	PC(38:5)	2.4
119	756.5540	756.5540	0.1	290.2	283.3	PC 34:3	PC(34:3)	2.4
120	780.5539	780.5542	0.3	294.4	287.3	PC 36:5	PC(36:5)	2.5
121	824.6164	824.6160	0.5	307.4	299.7	PC 39:4	PC(39:4)	2.6
122	568.3398	568.3397	0.2	242.5	236.4	LPC 22:6-SN2	LPC(22:6)>LPC(22:6/0:0)_and_LPC(0:0/22:6)	2.6
123	946.8796	946.8787	1.0	344.5	335.8	TG 18:1_18:1_21:0	TG(57:2) M + NH3	2.6
124	836.6160	836.6154	0.7	307.6	299.8	PC 40:5	PC(40:5)	2.6
125	647.5125	647.5122	0.5	267.9	275	SM d18:1_12:0	SM(d30:1)	-2.6
126	960.8952	960.8946	0.6	347.2	338.4	TG 18:1_18:1_22:0	TG(58:2) M + NH3	2.6
127	806.5694	806.5692	0.3	301.0	293.1	PC 38:6 (PC 16:0_22:6)	PC(38:6)	2.7
128	780.5903	780.5903	0.0	298.6	290.7	PC O-37:5	PC(O-37:5)	2.7
129	689.5593	689.5599	0.8	276.0	283.8	SM d33:1	SM(d33:1)	-2.8
130	930.8481	930.8477	0.4	340.0	330.9	TG 18:1_18:1_20:1	TG(56:3)>TG(18:1_18:1_20:1) M + NH3	2.8
131	944.7701	944.7694	0.8	335.7	326.7	TG 16:0_20:4_22:6	TG(58:10) M + NH3	2.8
132	690.6187	690.6187	0.1	285.7	294	CE 20:4	CE(20:4)M+NH3	-2.8
133	754.5383	754.5381	0.4	290.0	281.9	PC 34:4	PC(34:4)	2.9
134	731.6067	731.6062	0.7	284.1	292.6	SM d18:1_18:0	SM(d36:1)	-2.9
135	675.5438	675.5447	1.4	272.3	280.5	SM d32:1	SM(d32:1)	-2.9

136	848.6526	848.6528	0.2	313.1	304.2	PC O-42:6	PC(O-42:6)	2.9
137	508.3400	508.3400	0.1	225.6	232.5	LPC 17:1-SN2	LPC(17:1)>LPC(17:1/0:0)_and_LPC(0:0/17:1)	-3.0
138	866.6632	866.6643	1.4	318.2	308.8	PC 42:4	PC(42:4)	3.0
139	494.3242	494.3244	0.3	221.5	228.5	LPC 16:1-SN2	LPC(16:1)>LPC(16:1/0:0)_and_LPC(0:0/16:1)	-3.0
140	948.8956	948.8944	1.3	347.6	337.2	TG 16:0_16:1_25:0	TG(57:1)>TG(16:0_18:1_23:0)_and_TG(18:0_18:1_21:0) M + NH3	3.1
141	794.5695	794.5694	0.0	300.1	291.1	PC 37:5	PE(40:5)	3.1
142	522.3556	522.3562	1.1	227.9	235.2	LPC 18:1-SN1	LPC(18:1)>LPC(18:1/0:0)_and_LPC(0:0/18:1)	-3.1
143	568.3398	568.3396	0.5	242.6	235.2	LPC 22:6-SN1	LPC(22:6)>LPC(22:6/0:0)_and_LPC(0:0/22:6)	3.1
144	522.3555	522.3554	0.2	229.3	236.9	LPC 18:1-SN2	LPC(18:1)>LPC(18:1/0:0)_and_LPC(0:0/18:1)	-3.2
145	958.8795	958.8787	0.8	347.2	336.4	TG 18:1_18:2_22:0	TG(58:3) M + NH3	3.2
146	806.5695	806.5689	0.7	301.0	291.4	PC 38:6 (PC 18:2_20:4)	PC(38:6)	3.3
147	570.3554	570.3553	0.2	244.3	236.4	LPC 22:5-SN1	LPC(22:5)>LPC(22:5/0:0)_and_LPC(0:0/22:5)	3.3
148	703.5749	703.5778	4.2	276.6	286.3	SM d18:1_16:0	SM(d34:1)	-3.4
149	482.3242	482.3243	0.4	220.4	228.3	LPC 15:0-SN1	LPC(15:0)>LPC(15:0/0:0)_and_LPC(0:0/15:0)	-3.5
150	832.5845	832.5846	0.1	307.5	296.8	PC 40:7	PC(40:7)	3.6
151	705.5903	705.5891	1.6	279.5	290.1	SM d34:0	SM(d34:0)	-3.7
152	820.5848	820.5850	0.3	307.7	296.6	PC 39:6	PC(39:6)	3.8
153	677.5595	677.5582	1.9	274.2	285	SM d32:0	SM(d32:0)	-3.8
154	572.3710	572.3710	0.1	247.8	238.6	LPC 22:4-SN1	LPC(22:4)>LPC(22:4/0:0)_and_LPC(0:0/22:4)	3.8
155	864.6473	864.6473	0.0	318.3	305.5	PC 42:5	PC(42:5)	4.2
156	830.5693	830.5690	0.4	307.1	294	PC 20:4_20:4 Cis	PC(40:8)	4.4
157	524.3710	524.3717	1.2	228.9	240.1	LPC 18:0-SN1	LPC(18:0)>LPC(18:0/0:0)_and_LPC(0:0/18:0)	-4.7
158	774.6376	774.6368	1.0	283.0	297.4	PC O-36:1	PC(O-36:1)	-4.8

159	764.52271	764.52264	0.1	294.0	280	PE 16:0_22:6	PE(38:6)	5.0
160	496.33987	496.34047	1.2	220.5	232.1	LPC 16:0-SN1	LPC(16:0)>LPC(16:0/0:0)_and_LPC(0:0/16:0)	-5.0
161	524.37122	524.37145	0.4	228.4	241	LPC 18:0-SN2	LPC(18:0)>LPC(18:0/0:0)_and_LPC(0:0/18:0)	-5.2
162	468.30847	468.3084	0.1	212.4	224.6	LPC 14:0-SN1	LPC(14:0)>LPC(14:0/0:0)_and_LPC(0:0/14:0)	-5.4
163	510.35565	510.35546	0.4	223.1	236.4	LPC 17:0-SN1	LPC(17:0)>LPC(17:0/0:0)_and_LPC(0:0/17:0)	-5.6
164	468.3085	468.30869	0.4	212.5	225.5	LPC 14:0-SN2	LPC(14:0)>LPC(14:0/0:0)_and_LPC(0:0/14:0)	-5.8
165	496.33994	496.34021	0.5	218.2	233.7	LPC 16:0-SN2	LPC(16:0)>LPC(16:0/0:0)_and_LPC(0:0/16:0)	-6.6
166	766.57428	766.57459	0.4	307.0	287.5	PC O-36:5 (PC O-16:1_20:4)	PC(O-36:5)	6.8

Supplementary Table S8. Summary of lipid features with matched ^{Orbi}CCS to reference ^{DT}CCS dataset for experiments conducted at a resolution of 240,000 and turbopump speed 68%.

#	m/z_this study	m/z_database	mzError_ppm	^{Orbi}CCS_this study	^{DT}CCS_database	Name_database	Name_This study	% error
1	729.5905	729.5910	0.7	285.4	285.3	SM 36:2	SM(d36:2)	0.1
2	787.6688	787.6693	0.6	298.5	299.1	SM 40:1	SM(d40:1)	-0.2
3	538.3870	538.3872	0.4	240.0	240.5	LPC 19:0	LPC(19:0)>LPC(19:0/0:0)_and_LPC(0:0/19:0)	-0.2
4	786.6007	786.6012	0.7	294.2	293.3	PC (18:1/18:1) (del9-trans)	PC(36:2)	0.3
5	799.6690	799.6693	0.4	301.1	300.1	SM 41:2	SM(d41:2)	0.3
6	745.6220	745.6223	0.5	290.8	289.8	SM 37:1	SM(d37:1)	0.4
7	759.6375	759.6380	0.6	292.3	293.4	SM 38:1	SM(d38:1)	-0.4
8	785.6533	785.6536	0.4	298.5	296.8	SM 40:2	SM(d40:2)	0.6
9	801.6846	801.6849	0.4	300.4	302.3	SM 41:1	SM(d41:1)	-0.6
10	827.7001	827.7006	0.6	307.8	305.7	SM 43:2	SM(d43:2)	0.7
11	773.6534	773.6536	0.3	294.9	297	SM 39:1	SM(d39:1)	-0.7
12	813.6845	813.6849	0.6	304.5	302.2	SM 42:2	SM(d42:2)	0.8
13	811.6687	811.6693	0.7	304.6	300.8	SM 42:3	SM(d42:3)	1.3
14	731.6067	731.6067	0.0	284.1	288.4	SM 36:1	SM(d36:1)	-1.5
15	703.5749	703.5754	0.7	276.6	281.2	SM 34:1	SM(d34:1)	-1.6
16	522.3555	522.3559	0.8	229.3	233.2	LPC 18:1	LPC(18:1)>LPC(18:1/0:0)_and_LPC(0:0/18:1)	-1.7
17	788.6165	788.6169	0.5	294.3	289.4	PC 36:1	PC(36:1)	1.7
18	760.5850	760.5856	0.8	292.9	287.9	PC (18:1(9Z)/16:0)	PC(34:1)	1.7
19	730.5385	730.5387	0.3	283.4	278.4	PC (16:1/16:1) (del9-cis)	PC(32:2)	1.8
20	552.4026	552.4029	0.6	242.6	247	20:0 Lyso PC	LPC(20:0)>LPC(20:0/0:0)_and_LPC(0:0/20:0)	-1.8
21	732.5539	732.5543	0.6	283.4	277.6	PC 32:1	PC(32:1)	2.1
22	774.6009	774.6013	0.5	292.8	285.8	PC 35:1	PC(35:1)	2.5
23	786.6007	786.6012	0.7	294.2	287.1	PC 36:2	PC(36:2)	2.5
24	758.5693	758.5699	0.8	290.7	280.6	PC 34:2	PC(34:2)	3.6
25	468.3085	468.3090	1.1	212.5	220.8	LPC 14:0	LPC(14:0)>LPC(14:0/0:0)_and_LPC(0:0/14:0)	-3.8
26	756.5540	756.5543	0.4	290.2	278.2	PC 34:3	PC(34:3)	4.3
27	524.3712	524.3716	0.7	228.4	238.8	18:0 Lyso PC	LPC(18:0)>LPC(18:0/0:0)_and_LPC(0:0/18:0)	-4.3
28	728.5593	728.5594	0.2	286.5	273.5	PE (O-36:3)	PE(O-36:3)>PE(O-18:1/18:2)	4.8
29	496.3399	496.3403	0.7	218.2	231.4	16:0 Lyso PC	LPC(16:0)>LPC(16:0/0:0)_and_LPC(0:0/16:0)	-5.7
30	400.3421	400.3427	1.5	187.2	214.7	Carnitine 16:0	Car(16:0)	-12.8

31	344.2795	344.2782	3.7	172.1	199.5	Carnitine 12:0	Car(12:0)	-13.7

Supplementary Table S9. Summary of lipid features with matched ^{Orbi}CCS to reference ^{TIMS}CCS dataset for experiments conducted at resolution of 180,000 and turbopump speed 68%.

#	m/z_this study	m/z_database	mzError_ppm	^{Orbi}CCS_this study	^{TIMS}CCS_database	Name_database	Name_This study	% error
1	520.3399	520.3402	0.6	231.2	231.2	LPC 18:2-SN2	LPC(18:2)>LPC(18:2/0:0)_and_LPC(0:0/18:2)	0.0
2	848.7701	848.7708	0.9	315.3	315.3	TG 16:0_16:1_18:1	TG(50:2)>TG(16:0_16:1_18:1)_and_TG(14:0_18:1_18:1) M + NH3	0.0
3	746.5695	746.5697	0.3	286.8	286.7	PC 33:1	PC(33:1)	0.0
4	813.6845	813.6862	2.1	306.8	306.6	SM d18:1_24:1	SM(d42:2)	0.1
5	827.7000	827.6999	0.1	310.4	310.1	SM d43:2	SM(d43:2)	0.1
6	820.7390	820.7395	0.5	309.0	309.3	TG 14:0_16:1_18:1	TG(48:2)>TG(14:0_16:0_18:2)_and_TG(14:0_16:1_18:1) M + NH3	-0.1
7	716.5588	716.5588	0.1	282.7	282.4	PC O-32:2	PC(O-32:2)	0.1
8	732.5540	732.5543	0.4	283.7	283.3	PC 16:0_16:1	PC(32:1)	0.1
9	874.7858	874.7875	1.9	320.5	320	TG 16:0_18:1_18:2	TG(52:3)>TG(16:0_18:1_18:2)_and_TG(16:1_18:1_18:1) M + NH3	0.2
10	832.7389	832.7390	0.1	311.5	310.9	TG 15:0_16:1_18:2	TG(49:3) M + NH3	0.2
11	744.5903	744.5890	1.7	288.1	287.6	PC O-34:2	PC(O-34:2)	0.2
12	762.6009	762.5990	2.5	293.5	292.8	PC 18:0_16:0	PC(34:0)	0.2
13	799.6690	799.6687	0.3	302.0	302.8	SM d41:2	SM(d41:2)	-0.3
14	727.5753	727.5748	0.7	286.0	286.8	SM d36:3	SM(d36:3)	-0.3
15	792.7076	792.7083	0.8	304.2	303.3	TG 12:0_16:1_18:1	TG(46:2) M + NH3	0.3
16	768.5903	768.5895	1.0	292.6	291.7	PC O-36:4	PC(O-36:4)	0.3
17	785.6533	785.6531	0.2	300.3	301.2	SM d40:2	SM(d40:2)	-0.3
18	794.7233	794.7233	0.0	304.4	305.5	TG 14:0_16:0_16:1	TG(46:1)>TG(12:0_16:0_18:1)_and_TG(10:0_18:0_18:1)_and_TG(14:0_16:0_16:1) M + NH3	-0.3
19	774.6008	774.6011	0.3	293.4	292.4	PC 35:1	PC(35:1)	0.4
20	850.7858	850.7857	0.1	316.8	318	TG 16:0_16:0_18:1	TG(50:1)>TG(16:0_16:0_18:1)_M+NH3	-0.4
21	818.7233	818.7238	0.6	308.6	307.4	TG 16:1_16:1_16:1	TG(48:3) M + NH3	0.4

22	846.7545	846.7556	1.4	314.8	313.5	TG 16:1_16:0_18:2	TG(50:3) M + NH3	0.4
23	834.7545	834.7550	0.6	310.8	312.2	TG 15:0_16:1_18:1	TG(49:2) M + NH3	-0.4
24	715.5750	715.5748	0.2	285.3	286.6	SM d35:2	SM(d35:2)	-0.5
25	836.7703	836.7703	0.1	316.1	314.6	TG 15:0_16:0_18:1	TG(49:1)>TG(15:0_16:0_18:0)_and_TG(16:0_16:0_17:1)_and_TG(16:0_16:1_17:0) M + NH3	0.5
26	766.6921	766.6927	0.8	298.1	299.8	TG 12:0_16:0_16:1	TG(44:1)>TG(10:0_16:0_18:1) M + NH3	-0.6
27	788.6165	788.6167	0.3	297.1	295.4	PC 18:0_18:1	PC(36:1)	0.6
28	536.3713	536.3720	1.3	239.2	237.8	LPC 19:1-SN1	LPC(19:1)>LPC(19:1/0:0)_and_LPC(0:0/19:1)	0.6
29	796.7390	796.7391	0.2	305.9	307.8	TG 14:0_16:0_16:0	TG(46:0)>TG(14:0_16:0_16:0)_and_TG(12:0_16:0_18:0) M + NH3	-0.6
30	796.6216	796.6210	0.9	299.4	297.5	PC O-38:4	PC(O-38:4)	0.6
31	862.7858	862.7861	0.4	320.2	318.2	TG 16:0_17:1_18:1	TG(51:2)>TG(15:0_18:1_18:1)_and_TG(16:0_17:1_18:1)_and_TG(16:1_17:0_18:1) M + NH3	0.6
32	794.6057	794.6055	0.3	297.6	295.7	PC O-38:5	PC(O-38:5)	0.6
33	772.6217	772.6210	0.9	296.3	294.3	PC O-36:2	PC(O-36:2)	0.7
34	872.7701	872.7720	2.1	320.1	317.7	TG 16:0_18:2_18:2	TG(52:4)>TG(16:0_16:0_20:4)_and_TG(16:1_18:1_18:2)_and_TG(16:0_18:1_18:2) M + NH3	0.7
35	706.5378	706.5382	0.5	278.4	280.5	PC 30:0	PC(30:0)	-0.8
36	830.7236	830.7228	1.0	311.8	309.4	TG 15:1_16:1_18:2	TG(49:4) M + NH3	0.8
37	546.3555	546.3547	1.5	237.3	235.4	LPC 20:3-SN2	LPC(20:3)>LPC(20:3/0:0)_and_LPC(0:0/20:3)	0.8
38	678.5070	678.5068	0.3	273.0	275.2	PC 14:0_14:0	PC(28:0)	-0.8
39	770.6059	770.6059	0.1	294.2	291.8	PC O-36:3	PC(O-36:3)	0.8
40	717.5905	717.5905	0.0	287.4	289.8	SM d35:1	SM(d35:1)	-0.8
41	550.3867	550.3868	0.1	240.3	242.4	LPC 20:1-SN1	LPC(20:1)>LPC(20:1/0:0)_and_LPC(0:0/20:1)	-0.9
42	822.7546	822.7547	0.1	308.8	311.6	TG 14:0_16:0_18:1	TG(48:1)>TG(16:0_16:0_16:1)_and_TG(14:0_16:0_18:1) M + NH3	-0.9
43	757.6218	757.6216	0.3	292.9	295.6	SM d38:2	SM(d38:2)	-0.9

44	786.6014	786.6026	1.5	295.5	292.7	PC 18:0_18:2	PC(36:2)	1.0
45	746.6062	746.6054	1.1	288.2	291	PC O-34:1	PC(O-34:1)	-1.0
46	811.6687	811.6685	0.2	306.9	303.9	SM d42:3	SM(d42:3)	1.0
47	772.5851	772.5857	0.8	292.5	289.4	PC 35:2	PC(35:2)	1.1
48	844.7387	844.7396	1.0	315.0	311.6	TG 14:0_18:2_18:2	TG(50:4)>TG(16:1_16:1_18:2)_and_TG(16:1_16:1_18:2) M + NH3	1.1
49	839.6997	839.6997	0.1	314.0	310.6	SM d44:3	SM(d44:3)	1.1
50	784.5851	784.5867	2.1	294.4	291.1	PC 18:1_18:2	PC(36:3)	1.1
51	787.6690	787.6697	1.0	299.5	303	SM d40:1	SM(d40:1)	-1.1
52	580.4338	580.4346	1.3	256.9	253.9	LPC 22:0-SN1	LPC(22:0)>LPC(22:0/0:0)_and_LPC(0:0/22:0)	1.2
53	825.6843	825.6846	0.3	309.7	306	SM d43:3	SM(d43:3)	1.2
54	902.8169	902.8167	0.2	329.7	325.8	TG 18:1_18:1_18:1	TG(54:3)>TG(18:0_18:1_18:2)_and_TG(18:1/18:1/18:1)_and_TG(16:0_18:2_20:1) M + NH3	1.2
55	548.3712	548.3708	0.8	239.5	236.5	LPC 20:2-SN1	LPC(20:2)>LPC(20:2/0:0)_and_LPC(0:0/20:2)	1.2
56	870.7545	870.7554	1.1	320.2	316.2	TG 16:1_18:2_18:2	TG(52:5)>TG(16:0_18:2_18:3) M + NH3	1.3
57	760.5851	760.5870	2.5	293.4	289.7	PC 16:0_18:1	PC(34:1)	1.3
58	688.6029	688.6026	0.5	295.1	291.4	CE 20:5	CE(20:5)M+NH3	1.3
59	734.5696	734.5696	0.0	282.9	286.6	PC 16:0_16:0	PC(32:0)	-1.3
60	718.5747	718.5748	0.1	281.4	285.2	PC O-32:1	PC(O-32:1)	-1.3
61	810.6010	810.6011	0.2	300.6	296.7	PC 18:0_20:4	PC(38:4)	1.3
62	774.6373	774.6368	0.7	293.3	297.4	PC O-36:1	PC(O-36:1)	-1.4
63	758.5694	758.5718	3.2	290.4	286.4	PC 16:0_18:2	PC(34:2)	1.4
64	721.5858	721.5842	2.2	286.3	290.4	SM 34:0;3O	SM(d34:0-OH)	-1.4
65	759.6376	759.6373	0.4	293.9	298.2	SM d38:1	SM(d38:1)	-1.4
66	714.6188	714.6185	0.4	291.4	295.9	CE 22:6	CE(22:6)M+NH3	-1.5

67	729.5907	729.5904	0.4	285.3	289.7	SM d18:1_18:1	SM(d36:2)	-1.5
68	730.5385	730.5386	0.2	283.9	279.6	PC 32:2	PC(32:2)	1.5
69	809.6527	809.6523	0.5	306.3	301.5	SM d42:4	SM(d40:1) M + Na	1.6
70	858.7543	858.7544	0.1	320.0	314.9	TG 15:0_18:2_18:2	TG(51:4)>TG(16:1_17:1_18:2)_and_TG(15:0_18:2_18:2)_and_TG(15:1_18:1_18:2) M + NH3	1.6
71	801.6847	801.6854	0.9	301.5	306.5	SM d41:1	SM(d41:1)	-1.6
72	546.3555	546.3552	0.5	237.7	233.8	LPC 20:3-SN1	LPC(20:3)>LPC(20:3/0:0)_and_LPC(0:0/20:3)	1.7
73	704.5225	704.5225	0.1	281.9	277.2	PC 30:1	PC(30:1)	1.7
74	494.3242	494.3243	0.3	223.3	227.1	LPC 16:1-SN1	LPC(16:1)>LPC(16:1/0:0)_and_LPC(0:0/16:1)	-1.7
75	748.6220	748.6205	1.9	289.4	294.4	PC O-34:0	PC(O-34:0)	-1.7
76	868.7387	868.7387	0.0	320.2	314.8	TG 16:1_18:2_18:3	TG(52:6) M + NH3	1.7
77	773.6532	773.6531	0.1	295.7	300.8	SM d39:1	SM(d39:1)	-1.7
78	538.3869	538.3872	0.5	238.8	243.2	LPC 19:0-SN2	LPC(19:0)>LPC(19:0/0:0)_and_LPC(0:0/19:0)	-1.8
79	782.5694	782.5701	0.8	295.8	290.4	PC 16:0_20:4	PC(36:4)	1.9
80	900.8011	900.8010	0.1	329.9	323.8	TG 18:1_18:1_18:2	TG(54:4)>TG(18:1_18:1_18:2) M + NH3	1.9
81	522.3552	522.3562	1.8	230.7	235.2	LPC 18:1-SN1	LPC(18:1)>LPC(18:1/0:0)_and_LPC(0:0/18:1)	-1.9
82	816.6474	816.6468	0.8	306.8	300.8	PC 38:1	PC(38:1)	2.0
83	888.8012	888.8012	0.0	329.5	323.1	TG 17:0_18:1_18:2	TG(53:3)>TG(17:0_18:1_18:2)_and_TG(17:1_18:1_18:1)_and_TG(16:1_18:1_19:1)_and_TG(16:0_18:1_19:2) M + NH3	2.0
84	552.4027	552.4025	0.3	241.3	246.4	LPC 20:0-SN1	LPC(20:0)>LPC(20:0/0:0)_and_LPC(0:0/20:0)	-2.1
85	812.6165	812.6163	0.2	303.3	297	PC 38:3	PC(38:3)	2.1
86	544.3399	544.3399	0.1	237.6	232.6	LPC 20:4-SN1	LPC(20:4)>LPC(20:4/0:0)_and_LPC(0:0/20:4)	2.2
87	814.6320	814.6311	1.1	304.9	298.4	PC 38:2	PC(38:2)	2.2
88	673.5281	673.5277	0.6	270.9	276.9	SM d32:2	SM(d32:2)	-2.2

89	720.5905	720.5898	0.9	281.6	287.9	PC O-32:0	PC(O-32:0)	-2.2
90	904.8326	904.8316	1.1	334.8	327.7	TG 18:0_18:1_18:1	TG(54:2)>TG(16:0_18:1_20:1)_and_TG(18:0_18:1_18:1) M + NH3	2.2
91	482.3242	482.3243	0.2	223.3	228.3	LPC 15:0-SN1	LPC(15:0)>LPC(15:0/0:0)_and_LPC(0:0/15:0)	-2.2
92	890.8170	890.8168	0.2	332.1	324.9	TG 17:0_18:1_18:1	TG(53:2) M + NH3	2.2
93	770.5696	770.5696	0.0	294.7	288	PC 35:3	PC(35:3)	2.3
94	898.7857	898.7855	0.2	329.5	322	TG 18:1_18:2_18:2	TG(54:5)>TG(18:1_18:2_18:2)_and_TG(16:0_16:0_22:5)_and_TG(16:0_18:1_20:4) M + NH3	2.3
95	802.6323	802.6332	1.2	305.3	298.3	PC 37:1	PC(37:1)	2.3
96	701.5595	701.5615	2.9	276.8	283.5	SM d18:1_16:1	SM(d34:2)	-2.4
97	886.7856	886.7852	0.5	328.8	321.1	TG 17:1_18:1_18:2	TG(53:4)>TG(17:0_18:2_18:2)_and_TG(17:1_18:1_18:2) M + NH3	2.4
98	718.5383	718.5383	0.0	286.4	279.7	PC 17:0_14:1	PC(31:1)	2.4
99	518.3242	518.3242	0.0	231.6	226.1	LPC 18:3-SN1	LPC(18:3)>LPC(18:3/0:0)_and_LPC(0:0/18:3)	2.4
100	808.5848	808.5848	0.1	301.4	294.2	PC 38:5	PC(38:5)	2.4
101	728.5228	728.5231	0.4	283.9	277.1	PC 32:3	PC(32:3)	2.4
102	782.5694	782.5695	0.2	295.1	287.5	PC 18:2_18:2 Cis	PC(36:4)	2.6
103	834.6007	834.6002	0.6	307.4	299.5	PC 18:0_22:6	PC(40:6)	2.6
104	733.6217	733.6208	1.3	287.8	295.7	SM d36:0	SM(d36:0)	-2.7
105	838.6318	838.6315	0.3	309.9	301.8	PC 40:4	PC(40:4)	2.7
106	768.5538	768.5540	0.3	294.1	286.2	PC 35:4	PC(35:4)	2.8
107	756.5540	756.5540	0.1	291.2	283.3	PC 34:3	PC(34:3)	2.8
108	522.3555	522.3554	0.2	230.3	236.9	LPC 18:1-SN2	LPC(18:1)>LPC(18:1/0:0)_and_LPC(0:0/18:1)	-2.8
109	960.8951	960.8946	0.5	347.9	338.4	TG 18:1_18:1_22:0	TG(58:2) M + NH3	2.8
110	731.6065	731.6062	0.4	284.3	292.6	SM d18:1_18:0	SM(d36:1)	-2.8
111	824.6524	824.6529	0.7	311.4	302.8	PC O-40:4	PC(O-40:4)	2.8

112	896.7700	896.7699	0.0	329.5	320.4	TG 18:2_18:2_18:2	TG(54:6)>TG(16:0_18:2_20:4) M + NH3	2.8
113	661.5282	661.5282	0.0	269.8	277.7	SM d31:1	SM(d31:1)	-2.9
114	745.6220	745.6221	0.2	286.7	295.2	SM d37:1	SM(d37:1)	-2.9
115	780.5539	780.5542	0.3	295.7	287.3	PC 36:5	PC(36:5)	2.9
116	508.3400	508.3400	0.1	225.7	232.5	LPC 17:1-SN2	LPC(17:1)>LPC(17:1/0:0)_and_LPC(0:0/ 17:1)	-2.9
117	884.7694	884.7688	0.7	328.9	319.5	TG 17:1_18:2_18:2	TG(53:5)>TG(17:1_18:2_18:2) M + NH3	2.9
118	689.5593	689.5599	0.8	275.2	283.8	SM d33:1	SM(d33:1)	-3.0
119	806.5695	806.5692	0.3	302.1	293.1	PC 38:6 (PC 16:0_22:6)	PC(38:6)	3.1
120	568.3398	568.3397	0.2	243.7	236.4	LPC 22:6-SN2	LPC(22:6)>LPC(22:6/0:0)_and_LPC(0:0/ 22:6)	3.1
121	754.5383	754.5381	0.3	290.7	281.9	PC 34:4	PC(34:4)	3.1
122	894.7543	894.7539	0.4	328.5	318.5	TG 18:2_18:2_18:3	TG(54:7)>TG(16:0_16:1_22:6)_and_TG(16:0_18:2_20:5) M + NH3	3.1
123	864.8010	864.8019	1.0	331.3	320.8	TG 16:0_17:0_18:1	TG(51:1) M + NH3	3.3
124	572.3710	572.3710	0.1	246.5	238.6	LPC 22:4-SN1	LPC(22:4)>LPC(22:4/0:0)_and_LPC(0:0/ 22:4)	3.3
125	958.8795	958.8787	0.8	347.6	336.4	TG 18:1_18:2_22:0	TG(58:3) M + NH3	3.3
126	703.5750	703.5778	4.1	276.7	286.3	SM d18:1_16:0	SM(d34:1)	-3.4
127	675.5438	675.5447	1.4	271.0	280.5	SM d32:1	SM(d32:1)	-3.4
128	647.5126	647.5122	0.6	265.6	275	SM d18:1_12:0	SM(d30:1)	-3.4
129	946.8795	946.8787	0.9	347.3	335.8	TG 18:1_18:1_21:0	TG(57:2) M + NH3	3.4
130	820.6212	820.6209	0.3	309.2	298.9	PC O-40:6	PC(O-40:6)	3.4
131	690.6186	690.6187	0.1	283.8	294	CE 20:4	CE(20:4)M+NH3	-3.5
132	824.6163	824.6160	0.5	310.2	299.7	PC 39:4	PC(39:4)	3.5
133	818.6055	818.6057	0.2	308.5	298	PC O-40:7	PC(O-40:7)	3.5
134	834.6009	834.5999	1.2	307.8	297.3	PC 40:6	PC(40:6)	3.5
135	570.3554	570.3553	0.1	244.9	236.4	LPC 22:5-SN1	LPC(22:5)>LPC(22:5/0:0)_and_LPC(0:0/ 22:5)	3.6

136	705.5904	705.5891	1.8	279.7	290.1	SM d34:0	SM(d34:0)	-3.6
137	806.5695	806.5689	0.7	302.0	291.4	PC 38:6 (PC 18:2_20:4)	PC(38:6)	3.6
138	568.3399	568.3396	0.6	243.9	235.2	LPC 22:6-SN1	LPC(22:6)>LPC(22:6/0:0)_and_LPC(0:0/ 22:6)	3.7
139	740.5231	740.5227	0.6	288.4	278	PE 16:0_20:4	PE(36:4)	3.7
140	780.5904	780.5903	0.1	301.8	290.7	PC O-37:5	PC(O-37:5)	3.8
141	677.5595	677.5582	1.9	273.4	285	SM d32:0	SM(d32:0)	-4.1
142	832.5847	832.5846	0.1	308.9	296.8	PC 40:7	PC(40:7)	4.1
143	524.3710	524.3717	1.2	229.8	240.1	LPC 18:0-SN1	LPC(18:0)>LPC(18:0/0:0)_and_LPC(0:0/ 18:0)	-4.3
144	510.3556	510.3555	0.3	225.9	236.4	LPC 17:0-SN1	LPC(17:0)>LPC(17:0/0:0)_and_LPC(0:0/ 17:0)	-4.4
145	932.8639	932.8642	0.3	348.2	333.3	TG 18:1_18:1_20:0	TG(56:2) M + NH3	4.5
146	924.8012	924.8006	0.6	342.8	327.6	TG 18:1_18:1_20:4	TG(56:6)>TG(18:1_18:1_20:4)_and_TG(16:0_18:1_22:5)_and_TG(18:1_18:2_20: 3) M + NH3	4.6
147	876.8016	876.8023	0.8	336.8	321.9	TG 16:0_18:1_18:1	TG(52:2)>TG(16:0_18:1_18:1) M + NH3	4.6
148	866.6629	866.6643	1.6	323.1	308.8	PC 42:4	PC(42:4)	4.6
149	468.3085	468.3084	0.1	213.9	224.6	LPC 14:0-SN1	LPC(14:0)>LPC(14:0/0:0)_and_LPC(0:0/ 14:0)	-4.8
150	820.5847	820.5850	0.4	311.0	296.6	PC 39:6	PC(39:6)	4.8
151	524.3712	524.3715	0.4	229.2	241	LPC 18:0-SN2	LPC(18:0)>LPC(18:0/0:0)_and_LPC(0:0/ 18:0)	-4.9
152	948.8954	948.8944	1.1	354.0	337.2	TG 16:0_16:1_25:0	TG(57:1)>TG(16:0_18:1_23:0)_and_TG(18:0_18:1_21:0) M + NH3	5.0
153	496.3399	496.3402	0.7	221.8	233.7	LPC 16:0-SN2	LPC(16:0)>LPC(16:0/0:0)_and_LPC(0:0/ 16:0)	-5.1
154	468.3085	468.3087	0.4	214.0	225.5	LPC 14:0-SN2	LPC(14:0)>LPC(14:0/0:0)_and_LPC(0:0/ 14:0)	-5.1
155	830.5693	830.5690	0.4	309.1	294	PC 20:4_20:4 Cis	PC(40:8)	5.1
156	944.7700	944.7694	0.7	344.2	326.7	TG 16:0_20:4_22:6	TG(58:10) M + NH3	5.4
157	764.5227	764.5226	0.1	295.3	280	PE 16:0_22:6	PE(38:6)	5.5
158	496.3399	496.3405	1.2	219.3	232.1	LPC 16:0-SN1	LPC(16:0)>LPC(16:0/0:0)_and_LPC(0:0/ 16:0)	-5.5

159	864.6476	864.6473	0.3	322.8	305.5	PC 42:5	PC(42:5)	5.7
160	766.5742	766.5746	0.5	307.2	287.5	PC O-36:5 (PC O-16:1_20:4)	PC(O-36:5)	6.8
161	856.5850	856.5846	0.4	319.1	297.6	PC 42:9	PC(42:9)	7.2

Supplementary Table S10. Summary of lipid features with matched ^{Orbi}CCS to reference ^{DT}CCS dataset for experiments conducted at resolution of 180,000 and turbopump speed 68%.

#	m/z_this study	m/z_database	mzError_ppm	^{Orbi}CCS_this study	^{DT}CCS_database	Name_database	Name_This study	% error
1	729.5907	729.5910	0.4	285.3	285.3	SM 36:2	SM(d36:2)	0.0
2	787.6690	787.6693	0.4	299.5	299.1	SM 40:1	SM(d40:1)	0.2
3	759.6376	759.6380	0.6	293.9	293.4	SM 38:1	SM(d38:1)	0.2
4	801.6847	801.6849	0.3	301.5	302.3	SM 41:1	SM(d41:1)	-0.3
5	773.6534	773.6536	0.3	296.0	297	SM 39:1	SM(d39:1)	-0.3
6	799.6690	799.6693	0.4	302.0	300.1	SM 41:2	SM(d41:2)	0.6
7	538.3869	538.3872	0.6	238.8	240.5	LPC 19:0	LPC(19:0)>LPC(19:0/0:0)_and_LPC(0:0/19:0)	-0.7
8	786.6014	786.6012	0.3	295.5	293.3	PC (18:1/18:1) (del9-trans)	PC(36:2)	0.7
9	785.6533	785.6536	0.4	299.9	296.8	SM 40:2	SM(d40:2)	1.0
10	745.6220	745.6223	0.5	286.7	289.8	SM 37:1	SM(d37:1)	-1.1
11	522.3552	522.3559	1.3	230.7	233.2	LPC 18:1	LPC(18:1)>LPC(18:1/0:0)_and_LPC(0:0/18:1)	-1.1
12	827.7001	827.7006	0.6	309.9	305.7	SM 43:2	SM(d43:2)	1.4
13	731.6065	731.6067	0.3	284.3	288.4	SM 36:1	SM(d36:1)	-1.4
14	813.6845	813.6849	0.6	306.8	302.2	SM 42:2	SM(d42:2)	1.5
15	703.5750	703.5754	0.6	276.7	281.2	SM 34:1	SM(d34:1)	-1.6
16	808.5823	808.5832	1.1	302.3	296.9	PC (18:1/18:1) (del9-trans)	PC(36:2) M + Na	1.8
17	760.5851	760.5856	0.7	293.4	287.9	PC (18:1(9Z)/16:0)	PC(34:1)	1.9
18	730.5385	730.5387	0.3	283.9	278.4	PC (16:1/16:1) (del9-cis)	PC(32:2)	2.0
19	811.6687	811.6693	0.7	306.9	300.8	SM 42:3	SM(d42:3)	2.0
20	732.5540	732.5543	0.5	283.7	277.6	PC 32:1	PC(32:1)	2.2
21	552.4027	552.4029	0.5	241.3	247	20:0 Lyso PC	LPC(20:0)>LPC(20:0/0:0)_and_LPC(0:0/20:0)	-2.3
22	774.6008	774.6013	0.6	293.4	285.8	PC 35:1	PC(35:1)	2.7
23	788.6165	788.6169	0.6	297.1	289.4	PC 36:1	PC(36:1)	2.7
24	546.3533	546.3535	0.4	234.8	241.5	18:0 Lyso PC	LPC(20:3)>LPC(20:3/0:0)_and_LPC(0:0/20:3)	-2.8
25	786.6007	786.6012	0.7	295.5	287.1	PC 36:2	PC(36:2)	2.9
26	518.3220	518.3222	0.5	227.3	234.3	16:0 Lyso PC	LPC(18:3)>LPC(18:3/0:0)_and_LPC(0:0/18:3)	-3.0
27	468.3085	468.3090	1.1	214.0	220.8	LPC 14:0	LPC(14:0)>LPC(14:0/0:0)_and_LPC(0:0/14:0)	-3.1
28	809.6527	809.6512	1.9	306.3	297.2	SM 40:1	SM(d40:1) M + Na	3.1
29	758.5694	758.5699	0.7	290.4	280.6	PC 34:2	PC(34:2)	3.5

30	524.3712	524.3716	0.7	229.2	238.8	18:0 Lyso PC	LPC(18:0)>LPC(18:0/0:0)_and_LPC(0:0/18:0)	-4.0
31	496.3399	496.3403	0.9	221.8	231.4	16:0 Lyso PC	LPC(16:0)>LPC(16:0/0:0)_and_LPC(0:0/16:0)	-4.1
32	756.5540	756.5543	0.4	291.2	278.2	PC 34:3	PC(34:3)	4.7
33	742.5382	742.5387	0.7	287.9	273.5	PE (18:1/18:1) (del9-cis)	PC(33:3)	5.3
34	728.5593	728.5594	0.1	288.0	273.5	PE (O-36:3)	PE(O-36:3)>PE(O-18:1/18:2)	5.3
35	750.5430	750.5413	2.3	294.4	279	PE (O-36:3)	PE(O-36:3)>PE(O-18:2/18:1)	5.5
36	400.3421	400.3427	1.5	189.7	214.7	Carnitine 16:0	Car(16:0)	-11.6
37	344.2795	344.2782	3.8	171.8	199.5	Carnitine 12:0	Car(12:0)	-13.9

Supplementary Table S11. Summary of lipid features with matched ^{Orbi}CCS to reference ^{TIMS}CCS dataset for experiments conducted at a resolution of 120,000 and turbopump speed 68%.

#	m/z_this study	m/z_database	mzError_ppm	^{Orbi}CCS_this study	^{TIMS}CCS_database	Name_database	Name_This study	% error
1	820.7391	820.7395	0.5	309.3	309.3	TG 14:0_16:1_18:1	TG(48:2)>TG(14:0_16:0_18:2)_and_TG(14:0_16:1_18:1) M + NH3	0.0
2	715.5750	715.5748	0.2	286.7	286.6	SM d35:2	SM(d35:2)	0.0
3	520.3397	520.3405	1.4	229.2	229.1	LPC 18:2-SN1	LPC(20:5)>LPC(20:5/0:0)_and_LPC(0:0/20:5)	0.0
4	746.6062	746.6054	1.1	290.8	291	PC O-34:1	PC(O-34:1)	-0.1
5	536.3712	536.3720	1.5	237.6	237.8	LPC 19:1-SN1	LPC(19:1)>LPC(19:1/0:0)_and_LPC(0:0/19:1)	-0.1
6	864.8014	864.8019	0.6	321.1	320.8	TG 16:0_17:0_18:1	TG(51:1) M + NH3	0.1
7	878.8170	878.8164	0.6	324.3	324	TG 16:0_18:0_18:1	TG(52:1) M + NH3	0.1
8	785.6533	785.6531	0.2	301.7	301.2	SM d40:2	SM(d40:2)	0.2
9	706.5380	706.5382	0.3	280.0	280.5	PC 30:0	PC(30:0)	-0.2
10	787.6690	787.6697	1.0	302.4	303	SM d40:1	SM(d40:1)	-0.2
11	678.5071	678.5068	0.5	274.6	275.2	PC 14:0_14:0	PC(28:0)	-0.2
12	546.3559	546.3547	2.2	236.1	235.4	LPC 20:3-SN2	LPC(20:3)>LPC(20:3/0:0)_and_LPC(0:0/20:3)	0.3
13	746.5696	746.5697	0.2	287.6	286.7	PC 33:1	PC(33:1)	0.3
14	799.6690	799.6687	0.3	303.7	302.8	SM d41:2	SM(d41:2)	0.3
15	792.7078	792.7083	0.6	302.3	303.3	TG 12:0_16:1_18:1	TG(46:2) M + NH3	-0.3
16	850.7858	850.7857	0.2	316.8	318	TG 16:0_16:0_18:1	TG(50:1)>TG(16:0_16:0_18:1)_M+NH3	-0.4
17	745.6221	745.6221	0.1	293.9	295.2	SM d37:1	SM(d37:1)	-0.4
18	874.7858	874.7875	2.0	321.5	320	TG 16:0_18:1_18:2	TG(52:3)>TG(16:0_18:1_18:2)_and_TG(16:1_18:1_18:1) M + NH3	0.5
19	748.6220	748.6205	2.0	293.0	294.4	PC O-34:0	PC(O-34:0)	-0.5
20	727.5753	727.5748	0.8	285.3	286.8	SM d36:3	SM(d36:3)	-0.5
21	796.7388	796.7391	0.4	309.5	307.8	TG 14:0_16:0_16:0	TG(46:0)>TG(14:0_16:0_16:0)_and_TG(12:0_16:0_18:0) M + NH3	0.5
22	718.5747	718.5748	0.1	283.5	285.2	PC O-32:1	PC(O-32:1)	-0.6

23	862.7857	862.7861	0.5	320.2	318.2	TG 16:0_17:1_18:1	TG(51:2)>TG(15:0_18:1_18:1)_and_TG(16:0_17:1_18:1)_and_TG(16:1_17:0_18:1) M + NH3	0.6
24	848.7701	848.7708	0.8	317.4	315.3	TG 16:0_16:1_18:1	TG(50:2)>TG(16:0_16:1_18:1)_and_TG(14:0_18:1_18:1) M + NH3	0.7
25	822.7548	822.7547	0.1	309.4	311.6	TG 14:0_16:0_18:1	TG(48:1)>TG(16:0_16:0_16:1)_and_TG(14:0_16:0_18:1) M + NH3	-0.7
26	520.3399	520.3402	0.6	229.6	231.2	LPC 18:2-SN2	LPC(18:2)>LPC(18:2/0:0)_and_LPC(0:0/18:2)	-0.7
27	732.5540	732.5543	0.3	285.3	283.3	PC 16:0_16:1	PC(32:1)	0.7
28	744.5898	744.5890	1.1	289.7	287.6	PC O-34:2	PC(O-34:2)	0.7
29	818.7234	818.7238	0.5	309.7	307.4	TG 16:1_16:1_16:1	TG(48:3) M + NH3	0.7
30	580.4339	580.4346	1.2	252.0	253.9	LPC 22:0-SN1	LPC(22:0)>LPC(22:0/0:0)_and_LPC(0:0/22:0)	-0.7
31	716.5584	716.5588	0.5	284.5	282.4	PC O-32:2	PC(O-32:2)	0.7
32	801.6846	801.6854	1.0	304.1	306.5	SM d41:1	SM(d41:1)	-0.8
33	733.6223	733.6208	2.1	293.3	295.7	SM d36:0	SM(d36:0)	-0.8
34	813.6845	813.6862	2.1	309.2	306.6	SM d18:1_24:1	SM(d42:2)	0.8
35	846.7546	846.7556	1.2	316.2	313.5	TG 16:1_16:0_18:2	TG(50:3) M + NH3	0.9
36	546.3553	546.3552	0.2	235.9	233.8	LPC 20:3-SN1	LPC(20:3)>LPC(20:3/0:0)_and_LPC(0:0/20:3)	0.9
37	548.3712	548.3708	0.8	238.7	236.5	LPC 20:2-SN1	LPC(20:2)>LPC(20:2/0:0)_and_LPC(0:0/20:2)	0.9
38	834.7545	834.7550	0.7	315.2	312.2	TG 15:0_16:1_18:1	TG(49:2) M + NH3	1.0
39	872.7701	872.7720	2.1	320.8	317.7	TG 16:0_18:2_18:2	TG(52:4)>TG(16:0_16:0_20:4)_and_TG(16:1_18:1_18:2)_and_TG(16:0_18:1_18:2) M + NH3	1.0
40	734.5697	734.5696	0.1	283.8	286.6	PC 16:0_16:0	PC(32:0)	-1.0
41	544.3398	544.3397	0.3	236.3	233.8	LPC 20:4-SN2	LPC(20:4)>LPC(20:4/0:0)_and_LPC(0:0/20:4)	1.1
42	768.5902	768.5895	0.9	294.8	291.7	PC O-36:4	PC(O-36:4)	1.1
43	794.7234	794.7233	0.1	302.1	305.5	TG 14:0_16:0_16:1	TG(46:1)>TG(12:0_16:0_18:1)_and_TG(10:0_18:0_18:1)_and_TG(14:0_16:0_16:1) M + NH3	-1.1

44	721.5858	721.5842	2.3	287.1	290.4	SM 34:0;3O	SM(d34:0-OH)	-1.1
45	766.6920	766.6927	0.9	296.3	299.8	TG 12:0_16:0_16:1	TG(44:1)>TG(10:0_16:0_18:1) M + NH3	-1.2
46	760.5852	760.5870	2.3	293.1	289.7	PC 16:0_18:1	PC(34:1)	1.2
47	827.7000	827.6999	0.1	313.8	310.1	SM d43:2	SM(d43:2)	1.2
48	844.7388	844.7396	0.9	315.4	311.6	TG 14:0_18:2_18:2	TG(50:4)>TG(16:1_16:1_18:2)_and_TG(16:1_16:1_18:2) M + NH3	1.2
49	720.5905	720.5898	0.9	284.3	287.9	PC O-32:0	PC(O-32:0)	-1.2
50	758.5694	758.5718	3.2	290.0	286.4	PC 16:0_18:2	PC(34:2)	1.3
51	550.3868	550.3868	0.2	239.3	242.4	LPC 20:1-SN1	LPC(20:1)>LPC(20:1/0:0)_and_LPC(0:0/20:1)	-1.3
52	772.5851	772.5857	0.8	293.2	289.4	PC 35:2	PC(35:2)	1.3
53	870.7545	870.7554	1.1	320.4	316.2	TG 16:1_18:2_18:2	TG(52:5)>TG(16:0_18:2_18:3) M + NH3	1.3
54	832.7389	832.7390	0.1	315.1	310.9	TG 15:0_16:1_18:2	TG(49:3) M + NH3	1.3
55	544.3398	544.3399	0.0	235.8	232.6	LPC 20:4-SN1	LPC(20:4)>LPC(20:4/0:0)_and_LPC(0:0/20:4)	1.4
56	538.3870	538.3872	0.4	239.8	243.2	LPC 19:0-SN2	LPC(19:0)>LPC(19:0/0:0)_and_LPC(0:0/19:0)	-1.4
57	729.5908	729.5904	0.5	285.7	289.7	SM d18:1_18:1	SM(d36:2)	-1.4
58	773.6533	773.6531	0.3	296.5	300.8	SM d39:1	SM(d39:1)	-1.4
59	868.7388	868.7387	0.1	319.4	314.8	TG 16:1_18:2_18:3	TG(52:6) M + NH3	1.5
60	774.6371	774.6368	0.4	301.8	297.4	PC O-36:1	PC(O-36:1)	1.5
61	786.6010	786.6026	2.1	297.1	292.7	PC 18:0_18:2	PC(36:2)	1.5
62	858.7545	858.7544	0.1	319.8	314.9	TG 15:0_18:2_18:2	TG(51:4)>TG(16:1_17:1_18:2)_and_TG(15:0_18:2_18:2)_and_TG(15:1_18:1_18:2) M + NH3	1.5
63	836.7702	836.7703	0.2	319.5	314.6	TG 15:0_16:0_18:1	TG(49:1)>TG(15:0_16:0_18:0)_and_TG(16:0_16:0_17:1)_and_TG(16:0_16:1_17:0) M + NH3	1.6
64	757.6218	757.6216	0.3	291.0	295.6	SM d38:2	SM(d38:2)	-1.6
65	811.6686	811.6685	0.1	308.7	303.9	SM d42:3	SM(d42:3)	1.6

66	788.6166	788.6167	0.2	300.1	295.4	PC 18:0_18:1	PC(36:1)	1.6
67	842.7190	842.7231	4.9	315.7	310.8	TG 14:0_18:2_18:3	TG(50:5) M + NH3	1.6
68	839.6996	839.6997	0.2	315.6	310.6	SM d44:3	SM(d44:3)	1.6
69	770.6057	770.6059	0.2	296.5	291.8	PC O-36:3	PC(O-36:3)	1.6
70	784.5851	784.5867	2.0	295.8	291.1	PC 18:1_18:2	PC(36:3)	1.6
71	762.6010	762.5990	2.6	297.8	292.8	PC 18:0_16:0	PC(34:0)	1.7
72	518.3242	518.3242	0.0	230.0	226.1	LPC 18:3-SN1	LPC(18:3)>LPC(18:3/0:0)_and_LPC(0:0/ 18:3)	1.7
73	759.6378	759.6373	0.6	292.9	298.2	SM d38:1	SM(d38:1)	-1.8
74	701.5595	701.5615	2.8	278.1	283.5	SM d18:1_16:1	SM(d34:2)	-1.9
75	673.5282	673.5277	0.8	271.6	276.9	SM d32:2	SM(d32:2)	-1.9
76	772.6216	772.6210	0.8	300.0	294.3	PC O-36:2	PC(O-36:2)	1.9
77	825.6846	825.6846	0.1	311.9	306	SM d43:3	SM(d43:3)	1.9
78	902.8167	902.8167	0.0	332.1	325.8	TG 18:1_18:1_18:1	TG(54:3)>TG(18:1/18:1/18:1) M + NH3	1.9
79	800.6160	800.6174	1.8	301.5	295.5	PC 37:2	PC(37:2)	2.0
80	730.5385	730.5386	0.1	285.4	279.6	PC 32:2	PC(32:2)	2.1
81	704.5226	704.5225	0.1	283.1	277.2	PC 30:1	PC(30:1)	2.1
82	888.8013	888.8012	0.1	330.1	323.1	TG 17:0_18:1_18:2	TG(53:3)>TG(17:0_18:1_18:2)_and_TG(17:1_18:1_18:1)_and_TG(16:1_18:1_19: 1)_and_TG(16:0_18:1_19:2) M + NH3	2.2
83	717.5906	717.5905	0.2	283.3	289.8	SM d35:1	SM(d35:1)	-2.2
84	794.6051	794.6055	0.5	302.3	295.7	PC O-38:5	PC(O-38:5)	2.2
85	904.8326	904.8316	1.2	335.1	327.7	TG 18:0_18:1_18:1	TG(54:2)>TG(16:0_18:1_20:1)_and_TG(18:0_18:1_18:1) M + NH3	2.3
86	552.4027	552.4025	0.3	240.8	246.4	LPC 20:0-SN1	LPC(20:0)>LPC(20:0/0:0)_and_LPC(0:0/ 20:0)	-2.3
87	816.6475	816.6468	0.8	307.7	300.8	PC 38:1	PC(38:1)	2.3
88	689.5595	689.5599	0.6	277.3	283.8	SM d33:1	SM(d33:1)	-2.3

89	960.8950	960.8946	0.4	346.2	338.4	TG 18:1_18:1_22:0	TG(58:2) M + NH3	2.3
90	494.3242	494.3243	0.2	221.7	227.1	LPC 16:1-SN1	LPC(16:1)>LPC(16:1/0:0)_and_LPC(0:0/16:1)	-2.4
91	677.5598	677.5582	2.3	278.2	285	SM d32:0	SM(d32:0)	-2.4
92	900.8012	900.8010	0.3	331.5	323.8	TG 18:1_18:1_18:2	TG(54:4)>TG(18:1_18:1_18:2) M + NH3	2.4
93	898.7858	898.7855	0.3	329.9	322	TG 18:1_18:2_18:2	TG(54:5)>TG(18:1_18:2_18:2)_and_TG(16:0_16:0_22:5)_and_TG(16:0_18:1_20:4) M + NH3	2.5
94	522.3552	522.3562	1.9	229.2	235.2	LPC 18:1-SN1	LPC(18:1)>LPC(18:1/0:0)_and_LPC(0:0/18:1)	-2.6
95	808.5850	808.5848	0.1	301.8	294.2	PC 38:5	PC(38:5)	2.6
96	782.5694	782.5701	0.8	298.1	290.4	PC 16:0_20:4	PC(36:4)	2.7
97	810.6008	810.6011	0.4	304.7	296.7	PC 18:0_20:4	PC(38:4)	2.7
98	886.7856	886.7852	0.5	329.7	321.1	TG 17:1_18:1_18:2	TG(53:4)>TG(17:0_18:2_18:2)_and_TG(17:1_18:1_18:2) M + NH3	2.7
99	802.6323	802.6332	1.1	306.4	298.3	PC 37:1	PC(37:1)	2.7
100	494.3242	494.3244	0.3	222.1	228.5	LPC 16:1-SN2	LPC(16:1)>LPC(16:1/0:0)_and_LPC(0:0/16:1)	-2.8
101	890.8167	890.8168	0.1	334.1	324.9	TG 17:0_18:1_18:1	TG(53:2) M + NH3	2.8
102	568.3398	568.3397	0.3	243.1	236.4	LPC 22:6-SN2	LPC(22:6)>LPC(22:6/0:0)_and_LPC(0:0/22:6)	2.8
103	896.7701	896.7699	0.2	329.5	320.4	TG 18:2_18:2_18:2	TG(52:3)>TG(16:0_18:1_18:2)_and_TG(16:1_18:1_18:1) M + NH3	2.9
104	731.6065	731.6062	0.4	284.2	292.6	SM d18:1_18:0	SM(d36:1)	-2.9
105	714.6188	714.6185	0.4	287.3	295.9	CE 22:6	CE(22:6)M+NH3	-2.9
106	946.8793	946.8787	0.7	345.6	335.8	TG 18:1_18:1_21:0	TG(57:2) M + NH3	2.9
107	958.8792	958.8787	0.5	346.3	336.4	TG 18:1_18:2_22:0	TG(58:3) M + NH3	2.9
108	572.3710	572.3710	0.1	245.7	238.6	LPC 22:4-SN1	LPC(22:4)>LPC(22:4/0:0)_and_LPC(0:0/22:4)	3.0
109	728.5229	728.5231	0.3	285.4	277.1	PC 32:3	PC(32:3)	3.0
110	780.5902	780.5903	0.1	299.4	290.7	PC O-37:5	PC(O-37:5)	3.0
111	894.7543	894.7539	0.4	328.1	318.5	TG 18:2_18:2_18:3	TG(54:7)>TG(16:0_16:1_22:6)_and_TG(16:0_18:2_20:5) M + NH3	3.0

112	570.3555	570.3553	0.3	243.6	236.4	LPC 22:5-SN1	LPC(22:5)>LPC(22:5/0:0)_and_LPC(0:0/22:5)	3.0
113	703.5750	703.5778	4.0	277.6	286.3	SM d18:1_16:0	SM(d34:1)	-3.0
114	705.5904	705.5891	1.8	281.2	290.1	SM d34:0	SM(d34:0)	-3.1
115	796.6217	796.6210	0.9	306.7	297.5	PC O-38:4	PC(O-38:4)	3.1
116	884.7701	884.7688	1.4	329.6	319.5	TG 17:1_18:2_18:2	TG(53:5)>TG(17:1_18:2_18:2) M + NH3	3.2
117	814.6321	814.6311	1.2	307.9	298.4	PC 38:2	PC(38:2)	3.2
118	522.3555	522.3554	0.2	229.4	236.9	LPC 18:1-SN2	LPC(18:1)>LPC(18:1/0:0)_and_LPC(0:0/18:1)	-3.2
119	675.5438	675.5447	1.4	271.5	280.5	SM d32:1	SM(d32:1)	-3.2
120	756.5540	756.5540	0.0	292.6	283.3	PC 34:3	PC(34:3)	3.3
121	482.3243	482.3243	0.2	220.7	228.3	LPC 15:0-SN1	LPC(15:0)>LPC(15:0/0:0)_and_LPC(0:0/15:0)	-3.3
122	812.6165	812.6163	0.2	307.1	297	PC 38:3	PC(38:3)	3.4
123	782.5696	782.5695	0.1	297.3	287.5	PC 18:2_18:2 Cis	PC(36:4)	3.4
124	568.3399	568.3396	0.6	243.3	235.2	LPC 22:6-SN1	LPC(22:6)>LPC(22:6/0:0)_and_LPC(0:0/22:6)	3.5
125	824.6166	824.6160	0.8	310.1	299.7	PC 39:4	PC(39:4)	3.5
126	934.8792	934.8793	0.2	346.9	335.2	TG 16:0_18:1_22:0	TG(56:1) M + NH3	3.5
127	780.5540	780.5542	0.3	297.9	287.3	PC 36:5	PC(36:5)	3.7
128	766.5751	766.5747	0.5	300.3	289.5	PC O-36:5 (PC O-16:0_20:5)	PC(O-36:5)	3.7
129	768.5539	768.5540	0.2	296.9	286.2	PC 35:4	PC(35:4)	3.7
130	754.5384	754.5381	0.4	292.5	281.9	PC 34:4	PC(34:4)	3.8
131	820.5850	820.5850	0.0	308.1	296.6	PC 39:6	PC(39:6)	3.9
132	948.8951	948.8944	0.8	350.6	337.2	TG 16:0_16:1_25:0	TG(57:1)>TG(16:0_18:1_23:0)_and_TG(18:0_18:1_21:0) M + NH3	4.0
133	834.6007	834.6002	0.6	312.0	299.5	PC 18:0_22:6	PC(40:6)	4.2
134	924.8013	924.8006	0.7	341.2	327.6	TG 18:1_18:1_20:4	TG(56:6)>TG(18:1_18:1_20:4)_and_TG(16:0_18:1_22:5)_and_TG(18:1_18:2_20:3) M + NH3	4.2

135	524.3710	524.3717	1.2	229.8	240.1	LPC 18:0-SN1	LPC(18:0)>LPC(18:0/0:0)_and_LPC(0:0/ 18:0)	-4.3
136	718.5385	718.5383	0.3	291.8	279.7	PC 17:0_14:1	PC(31:1)	4.3
137	770.5694	770.5696	0.3	300.5	288	PC 35:3	PC(35:3)	4.3
138	690.6186	690.6187	0.1	280.7	294	CE 20:4	CE(20:4)M+NH3	-4.5
139	468.3085	468.3084	0.1	214.2	224.6	LPC 14:0-SN1	LPC(14:0)>LPC(14:0/0:0)_and_LPC(0:0/ 14:0)	-4.6
140	524.3713	524.3715	0.3	229.9	241	LPC 18:0-SN2	LPC(18:0)>LPC(18:0/0:0)_and_LPC(0:0/ 18:0)	-4.6
141	510.3556	510.3555	0.2	225.4	236.4	LPC 17:0-SN1	LPC(17:0)>LPC(17:0/0:0)_and_LPC(0:0/ 17:0)	-4.7
142	926.8172	926.8162	1.1	344.6	329.2	TG 18:0_18:1_20:4	TG(56:5)>TG(16:0_18:1_22:4) M + NH3	4.7
143	876.8016	876.8023	0.9	337.1	321.9	TG 16:0_18:1_18:1	TG(52:2)>TG(16:0_18:1_18:1) M + NH3	4.7
144	774.6008	774.6011	0.3	306.3	292.4	PC 35:1	PC(35:1)	4.8
145	468.3085	468.3087	0.5	214.5	225.5	LPC 14:0-SN2	LPC(14:0)>LPC(14:0/0:0)_and_LPC(0:0/ 14:0)	-4.9
146	806.5695	806.5689	0.8	305.7	291.4	PC 38:6 (PC 18:2_20:4)	PC(38:6)	4.9
147	496.3399	496.3405	1.2	220.7	232.1	LPC 16:0-SN1	LPC(16:0)>LPC(16:0/0:0)_and_LPC(0:0/ 16:0)	-4.9
148	818.6057	818.6057	0.1	313.2	298	PC O-40:7	PC(O-40:7)	5.1
149	848.6526	848.6528	0.2	319.9	304.2	PC O-42:6	PC(O-42:6)	5.1
150	944.7701	944.7694	0.7	343.6	326.7	TG 16:0_20:4_22:6	TG(58:10) M + NH3	5.2
151	866.6638	866.6643	0.6	325.0	308.8	PC 42:4	PC(42:4)	5.3
152	832.5848	832.5846	0.3	312.6	296.8	PC 40:7	PC(40:7)	5.3
153	820.6211	820.6209	0.2	315.0	298.9	PC O-40:6	PC(O-40:6)	5.4
154	496.3399	496.3402	0.7	220.4	233.7	LPC 16:0-SN2	LPC(16:0)>LPC(16:0/0:0)_and_LPC(0:0/ 16:0)	-5.7
155	824.6523	824.6529	0.7	321.5	302.8	PC O-40:4	PC(O-40:4)	6.2
156	830.5696	830.5690	0.7	312.5	294	PC 20:4_20:4 Cis	PC(40:8)	6.3
157	864.6475	864.6473	0.2	325.8	305.5	PC 42:5	PC(42:5)	6.7

158	856.5850	856.5846	0.4	321.5	297.6	PC 42:9	PC(42:9)	8.0

Supplementary Table S12. Summary of lipid features with matched ^{Orbi}CCS to reference ^{DT}CCS dataset for experiments conducted at a resolution of 120,000 and turbopump speed 68%.

#	m/z_this study	m/z_database	mzError_ppm	^{Orbi}CCS_this study	^{DT}CCS_database	Name_database	Name_This study	% error
1	729.5908	729.5910	0.3	285.7	285.3	SM 36:2	SM(d36:2)	0.1
2	759.6378	759.6380	0.3	292.9	293.4	SM 38:1	SM(d38:1)	-0.2
3	773.6533	773.6536	0.4	296.5	297	SM 39:1	SM(d39:1)	-0.2
4	538.3870	538.3872	0.4	239.8	240.5	LPC 19:0	LPC(19:0)>LPC(19:0/0:0)_and_LPC(0:0/19:0)	-0.3
5	801.6846	801.6849	0.3	304.1	302.3	SM 41:1	SM(d41:1)	0.6
6	787.6690	787.6693	0.4	302.4	299.1	SM 40:1	SM(d40:1)	1.1
7	799.6690	799.6693	0.4	303.6	300.1	SM 41:2	SM(d41:2)	1.2
8	703.5750	703.5754	0.6	277.6	281.2	SM 34:1	SM(d34:1)	-1.3
9	786.6010	786.6012	0.3	297.1	293.3	PC (18:1/18:1) (del9-trans)	PC(36:2)	1.3
10	745.6221	745.6223	0.3	293.9	289.8	SM 37:1	SM(d37:1)	1.4
11	731.6065	731.6067	0.3	284.2	288.4	SM 36:1	SM(d36:1)	-1.5
12	785.6534	785.6536	0.3	301.6	296.8	SM 40:2	SM(d40:2)	1.6
13	522.3552	522.3559	1.4	229.2	233.2	LPC 18:1	LPC(18:1)>LPC(18:1/0:0)_and_LPC(0:0/18:1)	-1.7
14	760.5852	760.5856	0.5	293.1	287.9	PC (18:1(9Z)/16:0)	PC(34:1)	1.8
15	813.6845	813.6849	0.5	309.2	302.2	SM 42:2	SM(d42:2)	2.3
16	730.5385	730.5387	0.3	285.4	278.4	PC (16:1/16:1) (del9-cis)	PC(32:2)	2.5
17	552.4027	552.4029	0.5	240.8	247	20:0 Lyso PC	LPC(20:0)>LPC(20:0/0:0)_and_LPC(0:0/20:0)	-2.5
18	827.7001	827.7006	0.6	313.7	305.7	SM 43:2	SM(d43:2)	2.6
19	811.6686	811.6693	0.8	308.7	300.8	SM 42:3	SM(d42:3)	2.6
20	732.5540	732.5543	0.4	285.3	277.6	PC 32:1	PC(32:1)	2.8
21	808.5822	808.5832	1.2	305.7	296.9	PC (18:1/18:1) (del9-trans)	PC(36:2) M + Na	3.0
22	468.3085	468.3090	1.1	214.2	220.8	LPC 14:0	LPC(14:0)>LPC(14:0/0:0)_and_LPC(0:0/14:0)	-3.0
23	786.6007	786.6012	0.6	296.0	287.1	PC 36:2	PC(36:2)	3.1
24	758.5694	758.5699	0.7	290.0	280.6	PC 34:2	PC(34:2)	3.4
25	788.6166	788.6169	0.4	300.1	289.4	PC 36:1	PC(36:1)	3.7
26	524.3713	524.3716	0.6	229.9	238.8	18:0 Lyso PC	LPC(18:0)>LPC(18:0/0:0)_and_LPC(0:0/18:0)	-3.7
27	780.5511	780.5519	1.0	295.0	284.2	PC 34:2	PC(34:2) M + Na	3.8
28	744.5543	744.5543	0.1	290.0	279.1	PE (18:1/18:1) (del9-cis)	PC(33:2)	3.9
29	496.3399	496.3403	0.9	220.4	231.4	16:0 Lyso PC	LPC(16:0)>LPC(16:0/0:0)_and_LPC(0:0/16:0)	-4.8

30	756.5540	756.5543	0.4	292.6	278.2	PC 34:3	PC(34:3)	5.2
31	728.5591	728.5594	0.4	288.0	273.5	PE (O-36:3)	PE(O-36:3)>PE(O-18:1/18:2)	5.3
32	806.5695	806.5675	2.4	305.6	288.2	PC 36:3	PC(36:3)	6.1
33	774.6008	774.6013	0.6	306.3	285.8	PC 35:1	PC(35:1)	7.2
34	400.3421	400.3427	1.4	188.6	214.7	Carnitine 16:0	Car(16:0)	-12.1
35	344.2795	344.2782	3.8	171.6	199.5	Carnitine 12:0	Car(12:0)	-14.0

Supplementary Table S13. Summary of lipid features with matched ^{Orbi}CCS to reference ^{TIMS}CCS dataset for experiments conducted at a resolution of 90,000 and turbopump speed 68%.

#	m/z_this study	m/z_database	mzError_ppm	^{Orbi}CCS_this study	^{TIMS}CCS_database	Name_database	Name_This study	% error
1	678.5070	678.5068	0.3	275.1	275.2	PC 14:0_14:0	PC(28:0)	0.0
2	834.7545	834.7550	0.6	312.4	312.2	TG 15:0_16:1_18:1	TG(49:2) M + NH3	0.1
3	878.8170	878.8164	0.7	323.7	324	TG 16:0_18:0_18:1	TG(52:1) M + NH3	-0.1
4	787.6688	787.6697	1.1	302.7	303	SM d40:1	SM(d40:1)	-0.1
5	520.3397	520.3405	1.5	229.3	229.1	LPC 18:2-SN1	LPC(20:5)>LPC(20:5/0:0)_and_LPC(0:0/20:5)	0.1
6	785.6533	785.6531	0.2	300.8	301.2	SM d40:2	SM(d40:2)	-0.1
7	536.3712	536.3720	1.6	238.2	237.8	LPC 19:1-SN1	LPC(19:1)>LPC(19:1/0:0)_and_LPC(0:0/19:1)	0.2
8	862.7858	862.7861	0.4	318.8	318.2	TG 16:0_17:1_18:1	TG(51:2)>TG(15:0_18:1_18:1)_and_TG(16:0_17:1_18:1)_and_TG(16:1_17:0_18:1) M + NH3	0.2
9	874.7858	874.7875	2.0	320.6	320	TG 16:0_18:1_18:2	TG(52:3)>TG(16:0_18:1_18:2)_and_TG(16:1_18:1_18:1) M + NH3	0.2
10	820.7390	820.7395	0.5	308.6	309.3	TG 14:0_16:1_18:1	TG(48:2)>TG(14:0_16:0_18:2)_and_TG(14:0_16:1_18:1) M + NH3	-0.2
11	846.7545	846.7556	1.3	314.2	313.5	TG 16:1_16:0_18:2	TG(50:3) M + NH3	0.2
12	746.5697	746.5697	0.0	285.9	286.7	PC 33:1	PC(33:1)	-0.3
13	818.7235	818.7238	0.4	308.3	307.4	TG 16:1_16:1_16:1	TG(48:3) M + NH3	0.3
14	848.7701	848.7708	0.8	314.2	315.3	TG 16:0_16:1_18:1	TG(50:2)>TG(16:0_16:1_18:1)_and_TG(14:0_18:1_18:1) M + NH3	-0.3
15	827.7000	827.6999	0.1	311.2	310.1	SM d43:2	SM(d43:2)	0.4
16	799.6690	799.6687	0.4	303.9	302.8	SM d41:2	SM(d41:2)	0.4
17	746.6063	746.6054	1.2	289.9	291	PC O-34:1	PC(O-34:1)	-0.4
18	732.5540	732.5543	0.3	284.4	283.3	PC 16:0_16:1	PC(32:1)	0.4
19	876.8015	876.8023	0.9	320.5	321.9	TG 16:0_18:1_18:1	TG(52:2)>TG(16:0_18:1_18:1) M + NH3	-0.4
20	733.6220	733.6208	1.7	294.4	295.7	SM d36:0	SM(d36:0)	-0.5
21	832.7390	832.7390	0.1	312.3	310.9	TG 15:0_16:1_18:2	TG(49:3) M + NH3	0.5
22	744.5898	744.5890	1.1	289.0	287.6	PC O-34:2	PC(O-34:2)	0.5

23	706.5380	706.5382	0.3	279.1	280.5	PC 30:0	PC(30:0)	-0.5
24	836.7702	836.7703	0.2	316.2	314.6	TG 15:0_16:0_18:1	TG(49:1)>TG(15:0_16:0_18:0)_and_TG(16:0_16:0_17:1)_and_TG(16:0_16:1_17:0) M + NH3	0.5
25	796.7390	796.7391	0.2	309.3	307.8	TG 14:0_16:0_16:0	TG(46:0)>TG(14:0_16:0_16:0)_and_TG(12:0_16:0_18:0) M + NH3	0.5
26	520.3398	520.3402	0.7	230.0	231.2	LPC 18:2-SN2	LPC(18:2)>LPC(18:2/0:0)_and_LPC(0:0/18:2)	-0.5
27	792.7076	792.7083	0.8	301.6	303.3	TG 12:0_16:1_18:1	TG(46:2) M + NH3	-0.5
28	718.5748	718.5748	0.0	283.4	285.2	PC O-32:1	PC(O-32:1)	-0.6
29	813.6845	813.6862	2.1	308.6	306.6	SM d18:1_24:1	SM(d42:2)	0.7
30	872.7702	872.7720	2.1	319.8	317.7	TG 16:0_18:2_18:2	TG(52:4)>TG(16:0_16:0_20:4)_and_TG(16:1_18:1_18:2)_and_TG(16:0_18:1_18:2) M + NH3	0.7
31	715.5749	715.5748	0.1	288.6	286.6	SM d35:2	SM(d35:2)	0.7
32	716.5583	716.5588	0.7	284.6	282.4	PC O-32:2	PC(O-32:2)	0.8
33	768.5903	768.5895	1.0	294.1	291.7	PC O-36:4	PC(O-36:4)	0.8
34	844.7388	844.7396	1.0	314.2	311.6	TG 14:0_18:2_18:2	TG(50:4)>TG(16:1_16:1_18:2)_and_TG(16:1_16:1_18:2) M + NH3	0.8
35	801.6847	801.6854	0.9	303.9	306.5	SM d41:1	SM(d41:1)	-0.9
36	727.5753	727.5748	0.7	284.3	286.8	SM d36:3	SM(d36:3)	-0.9
37	850.7858	850.7857	0.2	315.2	318	TG 16:0_16:0_18:1	TG(50:1)>TG(16:0_16:0_18:1)_M+NH3	-0.9
38	822.7546	822.7547	0.1	308.9	311.6	TG 14:0_16:0_18:1	TG(48:1)>TG(16:0_16:0_16:1)_and_TG(14:0_16:0_18:1) M + NH3	-0.9
39	858.7545	858.7544	0.1	317.7	314.9	TG 15:0_18:2_18:2	TG(51:4)>TG(16:1_17:1_18:2)_and_TG(15:0_18:2_18:2)_and_TG(15:1_18:1_18:2) M + NH3	0.9
40	720.5540	720.5540	0.0	285.7	283.1	PC 31:0	PC(31:0)	0.9
41	546.3558	546.3547	2.1	237.6	235.4	LPC 20:3-SN2	LPC(20:3)>LPC(20:3/0:0)_and_LPC(0:0/20:3)	0.9
42	748.6221	748.6205	2.1	291.5	294.4	PC O-34:0	PC(O-34:0)	-1.0
43	786.6007	786.6026	2.5	295.8	292.7	PC 18:0_18:2	PC(36:2)	1.0
44	550.3867	550.3868	0.1	239.9	242.4	LPC 20:1-SN1	LPC(20:1)>LPC(20:1/0:0)_and_LPC(0:0/20:1)	-1.0

45	870.7545	870.7554	1.1	319.6	316.2	TG 16:1_18:2_18:2	TG(52:5)>TG(16:0_18:2_18:3) M + NH3	1.1
46	788.6165	788.6167	0.2	298.6	295.4	PC 18:0_18:1	PC(36:1)	1.1
47	734.5698	734.5696	0.2	283.4	286.6	PC 16:0_16:0	PC(32:0)	-1.1
48	811.6686	811.6685	0.1	307.4	303.9	SM d42:3	SM(d42:3)	1.2
49	760.5850	760.5870	2.6	293.1	289.7	PC 16:0_18:1	PC(34:1)	1.2
50	774.6370	774.6368	0.3	300.9	297.4	PC O-36:1	PC(O-36:1)	1.2
51	745.6220	745.6221	0.1	291.7	295.2	SM d37:1	SM(d37:1)	-1.2
52	580.4340	580.4346	1.1	256.9	253.9	LPC 22:0-SN1	LPC(22:0)>LPC(22:0/0:0)_and_LPC(0:0/ 22:0)	1.2
53	784.5851	784.5867	2.1	294.6	291.1	PC 18:1_18:2	PC(36:3)	1.2
54	688.6027	688.6026	0.2	287.8	291.4	CE 20:5	CE(20:5)M+NH3	-1.2
55	538.3870	538.3872	0.2	240.1	243.2	LPC 19:0-SN2	LPC(19:0)>LPC(19:0/0:0)_and_LPC(0:0/ 19:0)	-1.3
56	772.5850	772.5857	0.9	293.2	289.4	PC 35:2	PC(35:2)	1.3
57	839.6997	839.6997	0.0	314.7	310.6	SM d44:3	SM(d44:3)	1.3
58	758.5693	758.5718	3.2	290.2	286.4	PC 16:0_18:2	PC(34:2)	1.3
59	902.8168	902.8167	0.2	330.3	325.8	TG 18:1_18:1_18:1	TG(54:3)>TG(18:0_18:1_18:2)_and_TG(18:1/18:1/18:1)_and_TG(16:0_18:2_20:1) M + NH3	1.4
60	544.3398	544.3397	0.2	237.1	233.8	LPC 20:4-SN2	LPC(20:4)>LPC(20:4/0:0)_and_LPC(0:0/ 20:4)	1.4
61	720.5905	720.5898	0.9	283.8	287.9	PC O-32:0	PC(O-32:0)	-1.4
62	482.3241	482.3245	0.7	220.1	223.3	LPE 18:0 SN1	LPC(15:0)>LPC(15:0/0:0)_and_LPC(0:0/ 15:0)	-1.4
63	809.6527	809.6523	0.4	305.8	301.5		SM(d40:1) M + Na	1.4
64	704.5226	704.5225	0.1	281.2	277.2	PC 30:1	PC(30:1)	1.4
65	546.3555	546.3552	0.6	237.3	233.8	LPC 20:3-SN1	LPC(20:3)>LPC(20:3/0:0)_and_LPC(0:0/ 20:3)	1.5
66	888.8013	888.8012	0.0	328.0	323.1	TG 17:0_18:1_18:2	TG(53:3)>TG(17:0_18:1_18:2)_and_TG(17:1_18:1_18:1)_and_TG(16:1_18:1_19: 1)_and_TG(16:0_18:1_19:2) M + NH3	1.5

67	762.6010	762.5990	2.6	297.3	292.8	PC 18:0_16:0	PC(34:0)	1.5
68	766.6921	766.6927	0.8	295.2	299.8	TG 12:0_16:0_16:1	TG(44:1)>TG(10:0_16:0_18:1) M + NH3	-1.5
69	721.5858	721.5842	2.2	285.9	290.4	SM 34:0;3O	SM(d34:0-OH)	-1.5
70	825.6845	825.6846	0.1	310.7	306	SM d43:3	SM(d43:3)	1.5
71	868.7389	868.7387	0.2	319.7	314.8	TG 16:1_18:2_18:3	TG(52:6) M + NH3	1.6
72	794.7235	794.7233	0.3	300.7	305.5	TG 14:0_16:0_16:1	TG(46:1)>TG(12:0_16:0_18:1)_and_TG(10:0_18:0_18:1)_and_TG(14:0_16:0_16:1) M + NH3	-1.6
73	729.5906	729.5904	0.3	285.1	289.7	SM d18:1_18:1	SM(d36:2)	-1.6
74	730.5384	730.5386	0.2	284.0	279.6	PC 32:2	PC(32:2)	1.6
75	900.8012	900.8010	0.3	329.0	323.8	TG 18:1_18:1_18:2	TG(54:4)>TG(18:1_18:1_18:2) M + NH3	1.6
76	794.6058	794.6055	0.5	300.5	295.7	PC O-38:5	PC(O-38:5)	1.6
77	770.6058	770.6059	0.0	296.6	291.8	PC O-36:3	PC(O-36:3)	1.6
78	548.3713	548.3708	0.9	240.4	236.5	LPC 20:2-SN1	LPC(20:2)>LPC(20:2/0:0)_and_LPC(0:0/20:2)	1.7
79	898.7857	898.7855	0.2	327.4	322	TG 18:1_18:2_18:2	TG(54:5)>TG(18:1_18:2_18:2)_and_TG(16:0_16:0_22:5)_and_TG(16:0_18:1_20:4) M + NH3	1.7
80	757.6218	757.6216	0.3	290.5	295.6	SM d38:2	SM(d38:2)	-1.7
81	772.6216	772.6210	0.8	299.5	294.3	PC O-36:2	PC(O-36:2)	1.8
82	673.5281	673.5277	0.7	272.0	276.9	SM d32:2	SM(d32:2)	-1.8
83	796.5847	796.5848	0.1	299.1	293.8	PC 37:4	PC(37:4)	1.8
84	494.3242	494.3243	0.2	223.0	227.1	LPC 16:1-SN1	LPC(16:1)>LPC(16:1/0:0)_and_LPC(0:0/16:1)	-1.8
85	552.4025	552.4025	0.0	241.8	246.4	LPC 20:0-SN1	LPC(20:0)>LPC(20:0/0:0)_and_LPC(0:0/20:0)	-1.9
86	886.7856	886.7852	0.4	327.2	321.1	TG 17:1_18:1_18:2	TG(53:4)>TG(17:0_18:2_18:2)_and_TG(17:1_18:1_18:2) M + NH3	1.9
87	518.3242	518.3242	0.1	230.4	226.1	LPC 18:3-SN1	LPC(18:3)>LPC(18:3/0:0)_and_LPC(0:0/18:3)	1.9
88	810.6008	810.6011	0.4	302.4	296.7	PC 18:0_20:4	PC(38:4)	1.9

89	677.5598	677.5582	2.3	279.4	285	SM d32:0	SM(d32:0)	-2.0
90	904.8324	904.8316	0.9	334.1	327.7	TG 18:0_18:1_18:1	TG(54:2)>TG(16:0_18:1_20:1)_and_TG(18:0_18:1_18:1) M + NH3	2.0
91	773.6532	773.6531	0.1	294.9	300.8	SM d39:1	SM(d39:1)	-2.0
92	896.7701	896.7699	0.2	326.8	320.4	TG 18:2_18:2_18:2	TG(54:6)>TG(16:0_18:2_20:4) M + NH3	2.0
93	890.8169	890.8168	0.1	331.7	324.9	TG 17:0_18:1_18:1	TG(53:2) M + NH3	2.1
94	759.6376	759.6373	0.4	291.9	298.2	SM d38:1	SM(d38:1)	-2.1
95	544.3399	544.3399	0.1	237.5	232.6	LPC 20:4-SN1	LPC(20:4)>LPC(20:4/0:0)_and_LPC(0:0/ 20:4)	2.1
96	816.6475	816.6468	0.9	307.2	300.8	PC 38:1	PC(38:1)	2.1
97	508.3400	508.3400	0.1	227.5	232.5	LPC 17:1-SN2	LPC(17:1)>LPC(17:1/0:0)_and_LPC(0:0/ 17:1)	-2.1
98	701.5595	701.5615	2.8	277.2	283.5	SM d18:1_16:1	SM(d34:2)	-2.2
99	796.6217	796.6210	0.9	304.1	297.5	PC O-38:4	PC(O-38:4)	2.2
100	960.8951	960.8946	0.5	346.0	338.4	TG 18:1_18:1_22:0	TG(58:2) M + NH3	2.2
101	494.3242	494.3244	0.3	223.3	228.5	LPC 16:1-SN2	LPC(16:1)>LPC(16:1/0:0)_and_LPC(0:0/ 16:1)	-2.3
102	782.5693	782.5701	0.9	297.1	290.4	PC 16:0_20:4	PC(36:4)	2.3
103	884.7701	884.7688	1.4	327.0	319.5	TG 17:1_18:2_18:2	TG(53:5)>TG(17:1_18:2_18:2) M + NH3	2.3
104	894.7543	894.7539	0.4	326.2	318.5	TG 18:2_18:2_18:3	TG(54:7)>TG(16:0_16:1_22:6)_and_TG(16:0_18:2_20:5) M + NH3	2.4
105	800.6160	800.6174	1.8	302.7	295.5	PC 37:2	PC(37:2)	2.4
106	717.5906	717.5905	0.1	282.5	289.8	SM d35:1	SM(d35:1)	-2.5
107	812.6165	812.6163	0.2	304.8	297	PC 38:3	PC(38:3)	2.6
108	958.8795	958.8787	0.8	345.4	336.4	TG 18:1_18:2_22:0	TG(58:3) M + NH3	2.7
109	780.5903	780.5903	0.0	298.5	290.7	PC O-37:5	PC(O-37:5)	2.7
110	522.3558	522.3562	0.7	228.6	235.2	LPC 18:1-SN1	LPC(18:1)>LPC(18:1/0:0)_and_LPC(0:0/ 18:1)	-2.8
111	647.5126	647.5122	0.6	267.2	275	SM d18:1_12:0	SM(d30:1)	-2.8
112	946.8795	946.8787	0.9	345.3	335.8	TG 18:1_18:1_21:0	TG(57:2) M + NH3	2.8

113	572.3711	572.3710	0.2	245.5	238.6	LPC 22:4-SN1	LPC(22:4)>LPC(22:4/0:0)_and_LPC(0:0/22:4)	2.9
114	744.5543	744.5541	0.2	288.8	280.6		PC(33:2)	2.9
115	689.5595	689.5599	0.6	275.5	283.8	SM d33:1	SM(d33:1)	-2.9
116	731.6065	731.6062	0.4	284.0	292.6	SM d18:1_18:0	SM(d36:1)	-2.9
117	814.6321	814.6311	1.2	307.2	298.4	PC 38:2	PC(38:2)	2.9
118	802.6322	802.6332	1.3	307.1	298.3	PC 37:1	PC(37:1)	3.0
119	675.5438	675.5447	1.4	272.1	280.5	SM d32:1	SM(d32:1)	-3.0
120	705.5905	705.5891	1.9	281.2	290.1	SM d34:0	SM(d34:0)	-3.1
121	714.6188	714.6185	0.5	286.8	295.9	CE 22:6	CE(22:6)M+NH3	-3.1
122	482.3242	482.3243	0.3	220.9	228.3	LPC 15:0-SN1	LPC(15:0)>LPC(15:0/0:0)_and_LPC(0:0/15:0)	-3.2
123	782.5694	782.5695	0.2	296.8	287.5	PC 18:2_18:2 Cis	PC(36:4)	3.2
124	522.3554	522.3554	0.1	229.2	236.9	LPC 18:1-SN2	LPC(18:1)>LPC(18:1/0:0)_and_LPC(0:0/18:1)	-3.3
125	808.5823	808.5848	3.2	303.9	294.2	PC 38:5	PC(36:2) M + Na	3.3
126	703.5749	703.5778	4.2	276.8	286.3	SM d18:1_16:0	SM(d34:1)	-3.3
127	780.5539	780.5542	0.3	297.0	287.3	PC 36:5	PC(36:5)	3.4
128	756.5541	756.5540	0.1	292.8	283.3	PC 34:3	PC(34:3)	3.4
129	568.3398	568.3397	0.2	244.7	236.4	LPC 22:6-SN2	LPC(22:6)>LPC(22:6/0:0)_and_LPC(0:0/22:6)	3.5
130	570.3554	570.3553	0.2	244.7	236.4	LPC 22:5-SN1	LPC(22:5)>LPC(22:5/0:0)_and_LPC(0:0/22:5)	3.5
131	824.6166	824.6160	0.8	310.3	299.7	PC 39:4	PC(39:4)	3.5
132	768.5538	768.5540	0.3	296.5	286.2	PC 35:4	PC(35:4)	3.6
133	820.5850	820.5850	0.0	307.5	296.6	PC 39:6	PC(39:6)	3.7
134	924.8013	924.8006	0.7	339.8	327.6	TG 18:1_18:1_20:4	TG(56:6)>TG(18:1_18:1_20:4)_and_TG(16:0_18:1_22:5)_and_TG(18:1_18:2_20:3) M + NH3	3.7
135	770.5695	770.5696	0.2	299.0	288	PC 35:3	PC(35:3)	3.8

136	806.5694	806.5692	0.3	304.4	293.1	PC 38:6 (PC 16:0_22:6)	PC(38:6)	3.8
137	754.5384	754.5381	0.4	292.8	281.9	PC 34:4	PC(34:4)	3.9
138	948.8952	948.8944	0.9	350.4	337.2	TG 16:0_16:1_25:0	TG(57:1)>TG(16:0_18:1_23:0)_and_TG(18:0_18:1_21:0) M + NH3	3.9
139	834.6007	834.6002	0.6	311.6	299.5	PC 18:0_22:6	PC(40:6)	4.0
140	932.8640	932.8642	0.2	346.7	333.3	TG 18:1_18:1_20:0	TG(56:2) M + NH3	4.0
141	568.3399	568.3396	0.6	244.9	235.2	LPC 22:6-SN1	LPC(22:6)>LPC(22:6/0:0)_and_LPC(0:0/22:6)	4.1
142	774.6008	774.6011	0.3	304.4	292.4	PC 35:1	PC(35:1)	4.1
143	690.6185	690.6187	0.2	281.8	294	CE 20:4	CE(20:4)M+NH3	-4.1
144	510.3555	510.3555	0.1	226.4	236.4	LPC 17:0-SN1	LPC(17:0)>LPC(17:0/0:0)_and_LPC(0:0/17:0)	-4.2
145	468.3085	468.3084	0.2	215.1	224.6	LPC 14:0-SN1	LPC(14:0)>LPC(14:0/0:0)_and_LPC(0:0/14:0)	-4.2
146	718.5383	718.5383	0.1	292.1	279.7	PC 17:0_14:1	PC(31:1)	4.4
147	806.5695	806.5689	0.7	304.3	291.4	PC 38:6 (PC 18:2_20:4)	PC(38:6)	4.4
148	496.3399	496.3405	1.1	221.7	232.1	LPC 16:0-SN1	LPC(16:0)>LPC(16:0/0:0)_and_LPC(0:0/16:0)	-4.5
149	468.3085	468.3087	0.4	215.4	225.5	LPC 14:0-SN2	LPC(14:0)>LPC(14:0/0:0)_and_LPC(0:0/14:0)	-4.5
150	524.3710	524.3717	1.3	229.2	240.1	LPC 18:0-SN1	LPC(18:0)>LPC(18:0/0:0)_and_LPC(0:0/18:0)	-4.5
151	930.8480	930.8477	0.2	346.3	330.9	TG 18:1_18:1_20:1	TG(56:3)>TG(18:1_18:1_20:1) M + NH3	4.7
152	818.6057	818.6057	0.0	312.1	298	PC O-40:7	PC(O-40:7)	4.7
153	848.6525	848.6528	0.3	318.7	304.2	PC O-42:6	PC(O-42:6)	4.8
154	866.6639	866.6643	0.4	323.5	308.8	PC 42:4	PC(42:4)	4.8
155	524.3713	524.3715	0.3	229.3	241	LPC 18:0-SN2	LPC(18:0)>LPC(18:0/0:0)_and_LPC(0:0/18:0)	-4.8
156	946.7856	946.7851	0.6	344.8	328.5	TG 18:1_20:4_20:4	TG(56:6)>TG(18:1_18:1_20:4)_and_TG(16:0_18:1_22:5)_and_TG(18:1_18:2_20:3) M + NH3	5.0
157	820.6210	820.6209	0.1	314.2	298.9	PC O-40:6	PC(O-40:6)	5.1
158	944.7701	944.7694	0.7	343.7	326.7	TG 16:0_20:4_22:6	TG(58:10) M + NH3	5.2

159	832.5845	832.5846	0.1	312.5	296.8	PC 40:7	PC(40:7)	5.3
160	496.3400	496.3402	0.4	220.9	233.7	LPC 16:0-SN2	LPC(16:0)>LPC(16:0/0:0)_and_LPC(0:0/16:0)	-5.5
161	864.6475	864.6473	0.2	323.6	305.5	PC 42:5	PC(42:5)	5.9
162	824.6524	824.6529	0.7	321.0	302.8	PC O-40:4	PC(O-40:4)	6.0
163	830.5695	830.5690	0.6	312.2	294	PC 20:4_20:4 Cis	PC(40:8)	6.2
164	864.8030	864.8019	1.2	340.9	320.8	TG 16:0_17:0_18:1	TG(51:1) M + NH3	6.3
165	856.5847	856.5846	0.1	320.2	297.6	PC 42:9	PC(42:9)	7.6
166	766.5748	766.5747	0.0	320.3	289.5	PC O-36:5 (PC O-16:0_20:5)	PC(O-36:5)	10.6

Supplementary Table S14. Summary of lipid features with matched ^{Orbi}CCS to reference ^{DT}CCS dataset for experiments conducted at a resolution of 90,000 and turbopump speed 68%.

#	m/z_this study	m/z_database	mzError_ppm	^{Orbi}CCS_this study	^{DT}CCS_database	Name_database	Name_This study	% error
1	729.5906	729.5910	0.5	285.1	285.3	SM 36:2	SM(d36:2)	-0.1
2	538.3870	538.3872	0.3	240.1	240.5	LPC 19:0	LPC(19:0)>LPC(19:0/0:0)_and_LPC(0:0/19:0)	-0.2
3	801.6847	801.6849	0.3	303.9	302.3	SM 41:1	SM(d41:1)	0.5
4	759.6376	759.6380	0.6	291.9	293.4	SM 38:1	SM(d38:1)	-0.5
5	745.6220	745.6223	0.4	291.7	289.8	SM 37:1	SM(d37:1)	0.7
6	773.6532	773.6536	0.5	294.9	297	SM 39:1	SM(d39:1)	-0.7
7	786.6007	786.6012	0.7	295.8	293.3	PC (18:1/18:1) (del9-trans)	PC(36:2)	0.8
8	787.6688	787.6693	0.6	302.7	299.1	SM 40:1	SM(d40:1)	1.2
9	799.6690	799.6693	0.4	303.9	300.1	SM 41:2	SM(d41:2)	1.3
10	785.6534	785.6536	0.2	301.0	296.8	SM 40:2	SM(d40:2)	1.4
11	731.6065	731.6067	0.3	284.0	288.4	SM 36:1	SM(d36:1)	-1.5
12	703.5749	703.5754	0.7	276.8	281.2	SM 34:1	SM(d34:1)	-1.6
13	522.3554	522.3559	0.9	229.2	233.2	LPC 18:1	LPC(18:1)>LPC(18:1/0:0)_and_LPC(0:0/18:1)	-1.7
14	760.5850	760.5856	0.8	293.1	287.9	PC (18:1(9Z)/16:0)	PC(34:1)	1.8
15	827.7001	827.7006	0.6	311.3	305.7	SM 43:2	SM(d43:2)	1.8
16	730.5384	730.5387	0.4	284.0	278.4	PC (16:1/16:1) (del9-cis)	PC(32:2)	2.0
17	552.4025	552.4029	0.7	241.8	247	20:0 Lyso PC	LPC(20:0)>LPC(20:0/0:0)_and_LPC(0:0/20:0)	-2.1
18	813.6845	813.6849	0.5	308.6	302.2	SM 42:2	SM(d42:2)	2.1
19	811.6686	811.6693	0.8	307.4	300.8	SM 42:3	SM(d42:3)	2.2
20	808.5823	808.5832	1.1	303.9	296.9	PC (18:1/18:1) (del9-trans)	PC(36:2) M + Na	2.4
21	732.5540	732.5543	0.4	284.4	277.6	PC 32:1	PC(32:1)	2.4
22	468.3085	468.3090	1.1	215.1	220.8	LPC 14:0	LPC(14:0)>LPC(14:0/0:0)_and_LPC(0:0/14:0)	-2.6
23	786.6007	786.6012	0.7	295.0	287.1	PC 36:2	PC(36:2)	2.8
24	788.6165	788.6169	0.5	298.6	289.4	PC 36:1	PC(36:1)	3.2
25	758.5693	758.5699	0.7	290.2	280.6	PC 34:2	PC(34:2)	3.4
26	744.5543	744.5543	0.0	288.8	279.1	PE (18:1/18:1) (del9-cis)	PC(33:2)	3.5
27	524.3713	524.3716	0.6	229.3	238.8	18:0 Lyso PC	LPC(18:0)>LPC(18:0/0:0)_and_LPC(0:0/18:0)	-4.0
28	496.3400	496.3403	0.6	220.9	231.4	16:0 Lyso PC	LPC(16:0)>LPC(16:0/0:0)_and_LPC(0:0/16:0)	-4.5

29	524.3710	524.3716	1.2	229.2	240.7	2-18:0 Lyso PC	LPC(18:0)>LPC(18:0/0:0)_and_LPC(0:0/18:0)	-4.8
30	756.5541	756.5543	0.3	292.8	278.2	PC 34:3	PC(34:3)	5.3
31	728.5593	728.5594	0.1	288.4	273.5	PE (O-36:3)	PE(O-36:3)>PE(O-18:1/18:2)	5.5
32	774.6008	774.6013	0.6	304.4	285.8	PC 35:1	PC(35:1)	6.5
33	400.3421	400.3427	1.4	190.7	214.7	Carnitine 16:0	Car(16:0)	-11.2
34	344.2795	344.2782	3.8	172.5	199.5	Carnitine 12:0	Car(12:0)	-13.5
